# SETDB1-like MET-2 promotes transcriptional silencing and development independently of its H3K9me-associated catalytic activity

**DOI:** 10.1038/s41594-021-00712-4

**Published:** 2022-01-31

**Authors:** Colin E. Delaney, Stephen P. Methot, Veronique Kalck, Jan Seebacher, Daniel Hess, Susan M. Gasser, Jan Padeken

**Affiliations:** 1 Friedrich Miescher Institute for Biomedical Research, Basel, Switzerland; 2 Faculty of Sciences, University of Basel, Basel, Switzerland; 3 Present Address: Fondation ISREC, Lausanne, Switzerland

**Keywords:** Epigenomics, Gene silencing

## Abstract

Transcriptionally silenced heterochromatin bearing methylation of histone H3 on lysine 9 (H3K9me) is critical for maintaining organismal viability and tissue integrity. Here we show that in addition to ensuring H3K9me, MET-2, the *Caenorhabditis elegans* homolog of the SETDB1 histone methyltransferase, has a noncatalytic function that contributes to gene repression. Subnuclear foci of MET-2 coincide with H3K9me deposition, yet these foci also form when MET-2 is catalytically deficient and H3K9me is compromised. Whereas *met-2* deletion triggers a loss of silencing and increased histone acetylation, foci of catalytically deficient MET-2 maintain silencing of a subset of genes, blocking acetylation on H3K9 and H3K27. In normal development, this noncatalytic MET-2 activity helps to maintain fertility. Under heat stress MET-2 foci disperse, coinciding with increased acetylation and transcriptional derepression. Our study suggests that the noncatalytic, focus-forming function of this SETDB1-like protein and its intrinsically disordered cofactor LIN-65 is physiologically relevant.

## Abstract (web summary)

Genetic and genome-wide analysis of a catalytically deficient SETDB1-like enzyme, MET-2, in *Caenorhabditis* *elegans* reveals that MET-2 promotes transcriptional silencing and fertility through both H3K9 methylation and focus formation, which blocks histone acetylation.

## Main

The proper segregation of active and inactive regions of the genome during differentiation is critical for the establishment and maintenance of tissue and genome integrity in eukaryotes. Heterochromatic regions of the genome are condensed and sequestered at the nuclear periphery or around nucleoli and are typically not transcribed^1,2^. Constitutive heterochromatin is associated with H3K9me, which is necessary for the repression of satellite repeats and transposable elements^3^. Tissue-specific genes can also be repressed by H3K9me (refs. ^4–6^). Finally, H3K9me mediates chromatin anchoring at the nuclear periphery^7^ and helps prevent interaction between euchromatic and heterochromatic compartments^8,9^. In mammals, the loss of H3K9me is associated with aging, cancer and the impaired maintenance of tissue integrity^10–13^.

The enzymes that establish and maintain heterochromatin form subnuclear clusters or foci in many species^14–19^. In budding yeast, a function has been attributed to these stable perinuclear foci containing the Silent Information Regulator complex: the clustering promotes histone deacetylation and contributes to the stability and spread of repressed chromatin^19^. In mammals, the H3K9 histone methyltransferases (HMTs) SUV39H and SETDB1 remain bound to clusters of heterochromatin throughout the cell cycle^20,21^. In the case of SUV39H, this localized accumulation of the HMT requires an N-terminal chromodomain, which recognizes H3K9me2/me3 and thereby sequesters the enzyme at its chromatin targets^21^. To date, however, no function was assigned to the localized retention of H3K9 HMTs.

Recently, heterochromatic focus formation has been proposed to depend on disordered protein domains and multivalent interactions that promote local phase separation, as exemplified by the H3K9me reader HP1 protein^22,23^. In *Caenorhabditis elegans*, however, foci of MET-2, the SETDB1 homolog responsible for H3K9me deposition, and its disordered domain cofactor LIN-65, remain stable despite downregulation of the worm HP1 homologs^14^. Moreover, MET-2, unlike SUV39H, has no chromodomain. We therefore set out to examine what drives the focal clustering of heterochromatin proteins such as MET-2, and the relationship of such foci to histone H3K9me.

**Fig. 1:**
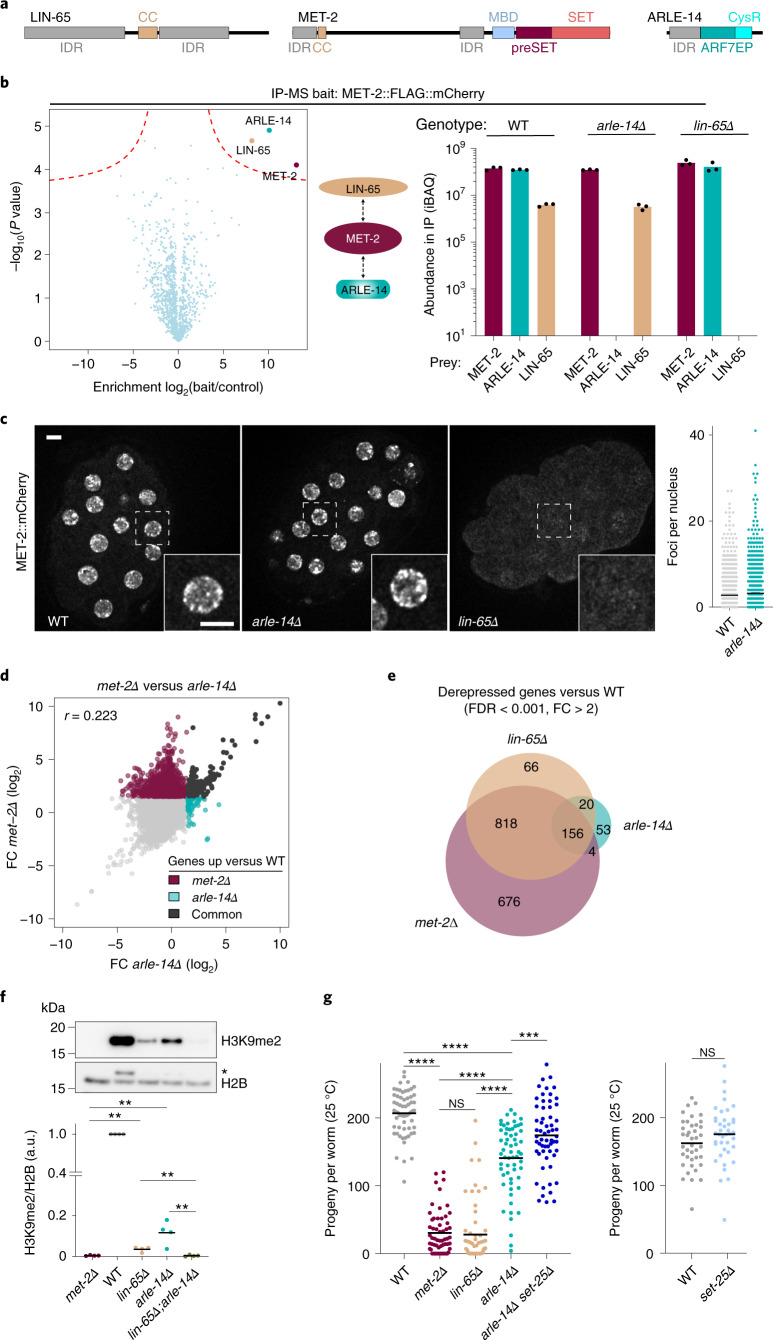
MET-2 foci promote efficient H3K9me and preserve fertility in H3K9me-deficient mutants. **a**, Domain layout of LIN-65, MET-2 and ARLE-14 proteins adapted from SMART(EMBL). IDR, intrinsically disordered region; CC, coiled-coil; CysR, cysteine-rich. **b**, Representative volcano plot from WT embryos and iBAQ (intensity-based absolute quantification) values for each indicated protein from immunoprecipitation–MS/MS using MET-2::FLAG::mCherry as bait. Bars indicate the mean and dots represent individual measurements. **c**, Representative live images of MET-2::mCherry in WT, *arle-14Δ(tm6845)* or *lin-65Δ* embryos and quantification of MET-2 foci. Scale bar, 5 µm. *N* = 3, *n* = 30. Nuclei: WT = 840, *arle-14Δ* = 961. **d**, Correlation between *met-2Δ* and *arle-14Δ* FC in gene expression (log_2_FC) compared with WT. Colors marking significantly changed genes (FDR < 0.001; log_2_FC > 1): *met-2*, dark red; *arle-14*, turquoise; common genes, black. Pearson correlation coefficient (*r*) is displayed. **e**, Venn diagram showing overlap of *lin-65Δ*-, *arle-14Δ*- and *met-2Δ*-derepressed genes. *lin-65Δ* data from ref. ^14^. **f**, Representative western blot and quantification of H3K9me2 normalized to histone H2B in WT, *lin-65;arle-14* embryos and sibling single mutants. *N* = 4. Image (*) marks residual H3K9me2 signal. *P*(*met-2Δ*, *lin-65Δ*) = 2.4 × 10^−3^, *P*(*met-2Δ*, *arle-14Δ*) = 9.0 × 10^−3^, *P*(*lin-65Δ*, *lin-65Δ;arle-14Δ*) = 2.6 × 10^−3^, *P*(*arle-14Δ, lin-65Δ;arle-14Δ*) = 9.0 × 10^−3^. **g**, Progeny number per worm at 25 °C in WT, *met-2Δ*, *lin-65Δ*, *arle-14Δ* and *arle-14Δ set-25Δ(n5021)*. *N* = 3, *n* = 60. Independent experiments comparing progeny number between WT and *set-25Δ* are included as a reference. *N* = 2, *n* = 40. *P*(WT, *met-2Δ*) = 6.7 × 10^−13^, *P*(WT, *arle-14Δ*) = 7.3 × 10^−13^, *P*(*met-2Δ, lin-65Δ*) = 0.998, *P*(*met-2Δ, arle-14Δ*) = 6.7 × 10^−13^, *P*(*lin-65Δ, arle-14Δ*) = 6.7 × 10^−13^, *P*(*arle-14Δ, arle-14Δ set-25Δ*) = 0.0002 by one-way analysis of variance (ANOVA), followed by Tukey post hoc test. ***P < 0.0001, ****P < 0.00001; a.u., arbitrary units; IP, immunoprecipitation. Source data

Although several *C. elegans* proteins have been implicated in methylation of H3K9 (refs. ^24–27^), only the double deletion of genes encoding two SET domain-containing proteins, MET-2 and SET-25, eliminates all detectable H3K9me, both in embryos^6,7,28^ and at later stages of somatic development^6,7,29^. The loss of H3K9me compromises the transcriptional repression of tissue-specific genes and repetitive elements^6,30^, although no other common histone methylation marks were altered upon *met-2* and *set-25* ablation^7^. The SET domain of MET-2 has 50% identity to that of SETDB1 (refs. ^18,28,31,32^), which has been shown to methylate histone H3K9 both in vivo^33^ and in vitro^34,35^. Moreover, MET-2 harbors a domain organization very similar to SETDB1, including a methyl CpG binding domain, Pre-SET domain and bifurcated SET domain at the C terminus (Fig. 1a). Nonetheless, the demonstration of HMT activity for recombinant MET-2 has been hindered because the purification of this large, partially unstructured protein with its cofactor, LIN-65, has proven difficult. We will therefore refer to MET-2 as a putative H3K9 HMT.

In cells, MET-2 and its two cofactors, the intrinsically disordered protein LIN-65 (refs. ^14,18^), and the poorly characterized but conserved ARLE-14, form foci that coincide with H3K9 methylation^14,18^. Not only the MET-2 partners, but also their regulatory interactions, are conserved in flies (Windei and CG14464 bind dSETDB1)^36,37^ and mammals (ATF7IP and ARLE14EP bind SETDB1)^38–40^. Ablation of *lin-65* (or *atf7ip*) leads to a loss of MET-2 (or SETDB1) in nuclei, respectively^14,18,41^, and in worms, *lin-65* deletion compromises the formation of MET-2 foci and derepresses a large subset of MET-2-sensitive targets^14,18^. Consistent with the notion that the unstructured domains present in LIN-65 contribute to MET-2 focus formation, these foci are sensitive to interventions that disrupt phase-separated condensates, such as treatment with 1,6-hexanediol and heat^14^.

Here we examined the mechanisms of MET-2-mediated heterochromatin silencing and its impact on development and fertility. Unexpectedly, we discovered a function for MET-2 protein that operates alongside its role in H3K9 methylation and correlates with focus formation. Restoring foci with a catalytic-deficient MET-2 maintained low acetylation levels on heterochromatin and rescued somatic developmental rates, mitigating inappropriate transcription and infertility. This was achieved in an ARLE-14-dependent manner. We argue that MET-2 promotes gene repression through both catalytic and noncatalytic functions, the latter coinciding with heterochromatic MET-2 focus formation.

## Results

To understand how MET-2 is regulated, we examined in greater detail its interaction with ARLE-14 and LIN-65 (refs. ^14,18^). LIN-65, similar to its homolog ATF7IP, is composed of two very large regions of disorder surrounding a coiled-coil domain (Fig. 1a). Reports in flies and mammals have shown this coiled-coil domain binds the N terminus of SETDB1 (refs. ^16,42^). ARL14EP, the mammalian homolog of ARLE-14, requires its conserved cysteine-rich C-terminal domain to bind SETDB1 (ref. ^43^). We examined the interdependence of MET-2 complex formation using mutants and mass spectrometry. In wild-type (WT, N2) embryos expressing MET-2::FLAG::mCherry, only ARLE-14 and LIN-65 were highly enriched in the MET-2 pulldown (Fig. 1b)^14,18^. Neither the ablation of *lin-65* (*lin-65Δ*(*gw1465*)) nor of *arle-14* (*arle-14Δ*(*tm6845*)) affected binding of the other cofactor to MET-2. We conclude that ARLE-14 and LIN-65 interact independently with MET-2.

**Extended Data Fig. 1:**
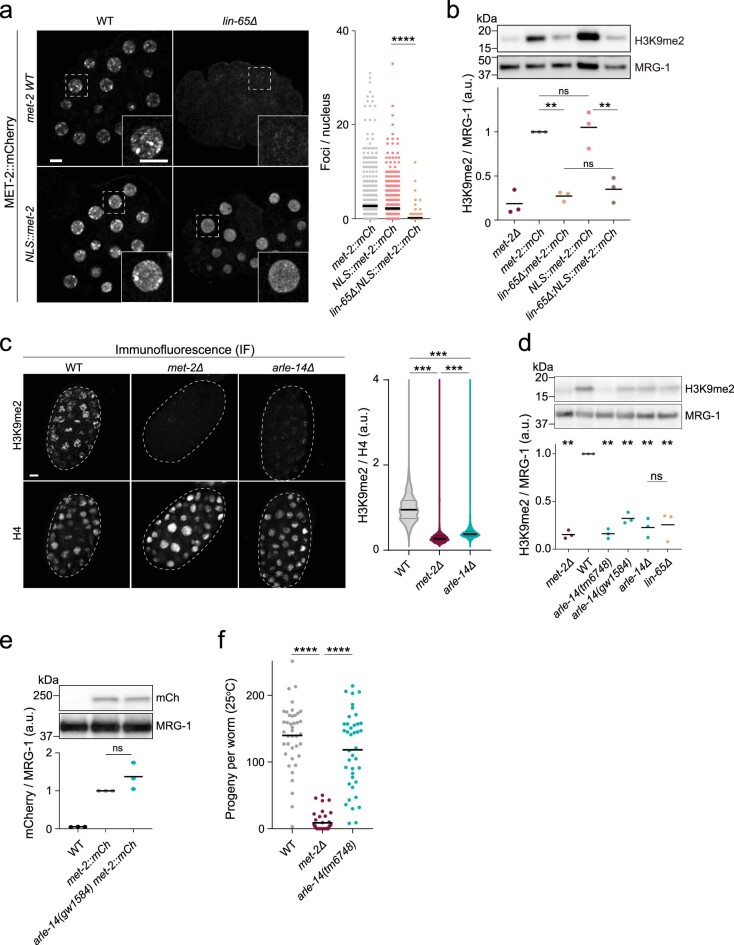
ARLE-14 acts in parallel with LIN-65 to promote H3K9me. (**a**) Representative images of live embryos expressing MET-2::mCherry from the endogenous *met-2* locus, either as wild-type (WT) or tagged with a nuclear localization sequence (NLS), + /- *lin-65Δ(gw1465)* deletion and quantification of MET-2 foci using automated image analysis. Foci number was not determinable in *lin-65*;*met-2::mCherry* due to the lack of MET-2::mCherry nuclear enrichment. Scale bar = 5 µm. N = 3, n = 30. Nuclei: *met-2::mCherry* = 700, *NLS::met-2::mCherry* = 594, *lin-65Δ;met-2::mCherry* = n.d., *lin-65Δ;met-2::mcherry* = 557. ****p-adj = 1.8*10^-11^ by one-way ANOVA, followed by Tukey post-hoc test. (**b**) Representative image of Western blot and quantification of H3K9me2 mean signal intensity normalized to MRG-1 in total cell extracts from strains shown in (A). N = 3. **p(*met-2::mCherry*, *lin-65Δ;met-2::mcherry*)=2.4*^10-5^, p(*NLS::met-2::mCherry*, *lin-65Δ;met-2::mCherry*)=8.7*10^-3^, ns = not significant by two-sided t-test. (**c**) Immunofluorescence and quantification of H3K9me2 intensity per nucleus normalized to H4 in embryos. Scale bar = 5 µm. N = 3, nuclei(embryos): WT = 885(37), *met-2Δ* = 985(51), and *arle-14(tm6845)* = 826(44). Median and quartiles shown. ***p-adj < 2.0*10^-16^ by one-way ANOVA, followed by Tukey post-hoc test. (**d**) Representative western blot and quantification of H3K9me2 in three independent *arle-14* mutants, *met-2Δ*, and *lin-65Δ*. N = 3. Statistics measure each mutant compared to WT. **p vs WT: *met-2Δ* = 4.5*10^-6^, *arle-14(tm6748)*=3.4*10^-5^, *arle-14(gw1584)*=3.4*10^-5^, *arle-14(tm6845)*=1.8*10^-4^, *lin-65Δ* = 1.1*10^-3^, ns = not significant by two-sided t-test. (**e**) Representative Western blot and quantification of MET-2::mcherry in WT and *arle-14(gw1584)* embryos. (-) indicates no mCherry tag present. N = 3. ns = not signficant by two-sided t-test. (**f**) Number of progeny per worm at 25 °C comparing *arle-14(tm6748)* to WT and *met-2Δ*. N = 2, n = 40. p(WT, met-2Δ)=1.34*10^-14^, p(met-2Δ, arle-14(tm6748))=2.8*10^-14^ by one-way ANOVA, followed by Tukey post-hoc test. Source data

Loss of *lin-65* impairs both MET-2 nuclear accumulation and concentration into subnuclear foci (Fig. 1c)^41^, and thus largely phenocopies *met-2* deletion^14,18^. To distinguish roles of nuclear accumulation from focus formation, we introduced a nuclear localization signal (NLS) to the endogenously tagged *met-2::mCherry* locus by CRISPR (*NLS::met-2*). The NLS::MET-2::mCherry nucleoplasmic signal was stronger than the WT MET-2::mCherry that traffics between the cytosol and nucleus^14,18^, but in both cases foci were clearly distinguishable (Extended Data Fig. 1a). Whereas the ablation of *lin-65* eliminated the nuclear enrichment of WT MET-2::mCherry, the NLS::MET-2 fusion protein remained nuclear but failed to form foci in the absence of LIN-65. Western blotting confirmed that H3K9me2 levels were not altered by the NLS tag on MET-2 as long as LIN-65 was present, and dropped similarly upon deletion of *lin-65* (Extended Data Fig. 1b). We conclude that nuclear accumulation of MET-2 is not sufficient for H3K9 HMT activity and focus formation, and that LIN-65 promotes both.

In contrast to LIN-65, ARLE-14 is not required for general silencing of repetitive elements^14,18^, and its loss compromised neither the nuclear import of MET-2 nor the number of foci formed (Fig. 1c). Nevertheless, embryos carrying either *arle-14Δ* or two other independent *arle-14* mutant alleles lost a majority of H3K9me2, as shown by quantitative fluorescence and western blots (Extended Data Fig. 1c,d). The impacts of *lin-65Δ* and of *arle-14Δ* on H3K9me2 levels in embryos were similar. Whereas we previously found that MET-2 stability is reduced in embryos lacking *lin-65* (ref. ^14^), the loss of *arle-14* did not reduce MET-2::mCherry levels (Extended Data Fig. 1e). These data argue that both ARLE-14 and LIN-65 promote MET-2 methylation activity, while only LIN-65 is essential for MET-2 focus formation.

Because the loss of H3K9me is associated with a failure to silence heterochromatin^5,6,33,44^, we examined gene expression in *arle-14Δ* using RNA sequencing (RNA-seq). Remarkably, only 14% of the genes derepressed by loss of *met-2* (*met-2Δ* genes) were upregulated in *arle-14Δ* (Fig. 1d,e), while 59% of the *met-2Δ*-derepressed genes were upregulated in *lin-65Δ*. These data suggested that residual low levels of H3K9me2 in combination with the preserved accumulation of MET-2 into foci (promoted by LIN-65 in the *arle-14* mutant) restricts gene derepression.

Because *arle-14* and *lin-65* mutants had different effects on transcription and focus formation, we performed epistasis analysis for the two genes, monitoring H3K9 methylation. We found that the *lin-65Δ;arle-14Δ* double mutant displayed a greater loss of H3K9me2 than either single mutant alone, achieving the same drop in methylation as in *met-2Δ* embryos (Fig. 1f). Consistent with the fact that ARLE-14 and LIN-65 interact with MET-2 independently of each other, we conclude that they function in parallel to promote MET-2’s role in H3K9 methylation.

*C. elegans* animals lacking all H3K9me reproduced efficiently when cultivated at 20 °C or below, yet the loss of *met-2* strongly accentuated the reduced fertility of worms at 25 °C, coincident with elevated levels of p53-dependent germline apoptosis (refs. ^6,30^). This loss of germline arises largely from the formation of R-loops at repeat elements, which are promiscuously transcribed upon loss of *met-2* (refs. ^6,30^). To test whether ARLE-14 and LIN-65 differentially affect worm fertility, we quantified the number of progeny in *arle-14Δ* and *lin-65Δ* mutants. We observed an enhanced loss of viable progeny in both *met-2Δ* and *lin-65Δ* animals at 25 °C, while the *arle-14Δ* population was only modestly decreased in comparison with WT worms, despite having a near equal drop in H3K9me2 (Fig. 1g and Extended Data Fig. 1f). The striking difference in the impact of *lin-65Δ* and *arle-14Δ* on fertility was substantiated independently at 20 °C (ref. ^14^, and see Fig. 4a below). The residual fertility in *arle-14Δ* animals was not dependent on SET-25, which is required for all H3K9me3 in embryos^7,28,30^, as the *arle-14Δ set-25Δ* double mutant was no less fertile than *arle-14Δ* alone (Fig. 1g).

We note that in both *lin-65Δ* and *arle-14Δ* embryos substantially more H3K9me2 is retained than in *met-2Δ* animals (Fig. 1f and Extended Data Fig. 1d), yet *lin-65Δ* phenotypes parallel those of *met-2Δ* more closely than those of *arle-14Δ*. This suggested that H3K9me2 and *met-2Δ* might not correlate strictly for all phenotypic read-outs, raising the possibility that MET-2 has functions beyond H3K9me deposition. These functions nonetheless require LIN-65 and its incumbent focus formation.

### Catalytically deficient MET-2 attenuates defects of *met-2Δ*

**Fig. 2:**
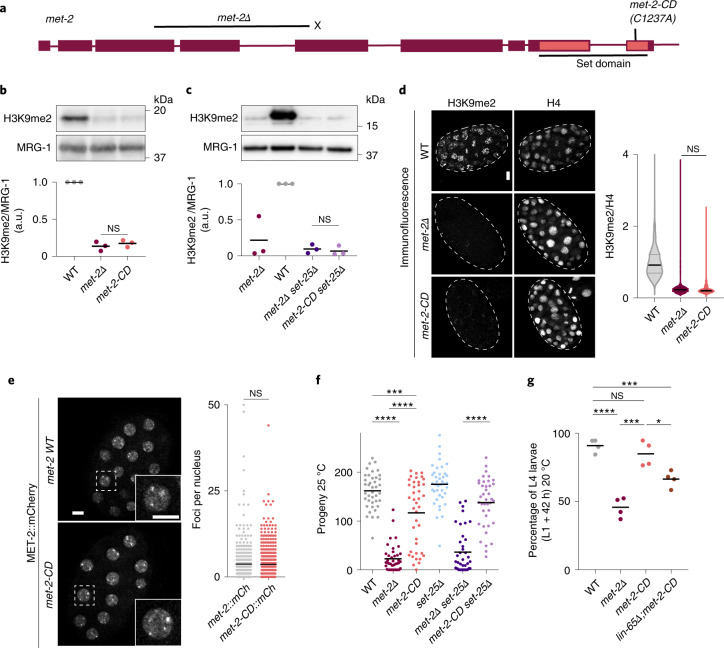
MET-2 deficient in H3K9 HMT activity forms foci and promotes germline and somatic development. **a**, Allele map of *met-2* locus showing deletion allele *met-2Δ(n4256)* as black bar and point mutant *met-2-CD(gw1660)*. Amino acid change is shown in parentheses. Putative stop codon for *met-2Δ* is shown with an ‘X’. The SET domain is indicated with light red. **b**,**c**, Representative western blot and quantification of H3K9me2 in WT, *met-2Δ* and *met-2-CD* (**b**) or WT, *met-2Δ*, *met-2Δ set-25Δ* and *met-2-CD set-25Δ* (**c**). *N* = 3. NS, nonsignificant by two-sided *t*-test. **d**, Immunofluorescence and quantification of H3K9me2 normalized to H4 in WT, *met-2Δ* and *met-2-CD*. Scale bar, 5 µm. *N* = 3, *n* nuclei(embryos): WT = 1,542(49), *met-2Δ* = 985(51), *met-2-CD* = 819(39). Median and quartiles shown. NS, not significant by one-way ANOVA, followed by Tukey post hoc test. **e**, Live imaging of embryos expressing either WT MET-2 or MET-2-CD::mCherry and quantification of MET-2 foci per nucleus. Scale bar, 5 µm. *N* = 3, nuclei(embryos): *met-2::mCherry* = 569(30), *met-2-CD::mCherry* = 613(30). NS, not significant by two-sided Wilcoxon signed-rank test. **f**, Brood sizes of the indicated strains at 25 °C. *N* = 2, *n* = 40. *P*(WT, *met-2Δ*) = 4.7 × 10^−14^, *P*(WT, *met-2-CD*) = 0.0002, *P*(*met-2-CD, met-2Δ*)=1.04 × 10^−13^, *P*(*met-2Δ set-25Δ, met-2-CD set-25Δ*) = 1.05 × 10^−13^, by one-way ANOVA, followed by Tukey post hoc test. **g**, Percentage of synchronized L1s that develop into L4 larvae 42 h after re-feeding at 20 °C. *N* = 4. *P*(WT, *met-2Δ*) = 4.7 × 10^−5^, *P*(WT, *lin-65Δ;met-2-CD*) = 7.2 × 10^−4^, *P*(*met-2Δ*, *met-2-CD*) = 5.5 × 10^−4^, *P*(*met-2-CD*, *lin-65Δ;met-2-CD*) = 0.015; ***P < 0.0001, ****P < 0.00001. Source data

**Extended Data Fig. 2:**
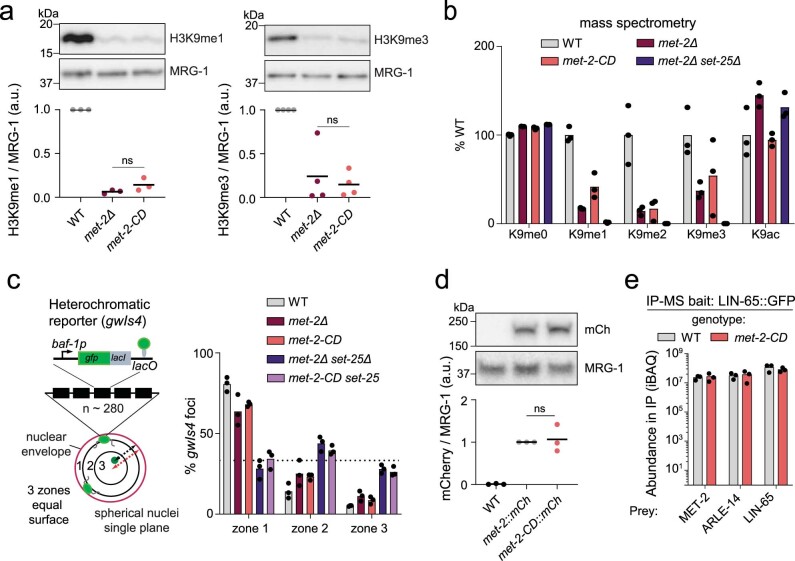
MET-2-CD deficient in HMT activity is stable in expression and interactions. (**a**) Representative Western blot and quantification of H3K9me1 and H3K9me3 in WT, *met-2Δ*, and *met-2-CD*. H3K9me1 N = 3, H3K9me3 N = 4. ns=non-significant by two-sided t-test. (**b**) Percentage of H3K9me1/me2/me3 and H3K9ac level in WT, *met-2Δ, met-2-CD* and *met-2 set-25* relative to WT detected by mass spectrometry normalized to the total H3K9 peptides detected. Bars indicate the mean and dots represent individual measurements. N = 3. (**c**) Scheme of the *gwIs4* heterochromatic reporter and zoning assay with quantification of array position in the indicated genotypes. We selected a single plane from a 3D stack in which the GFP signal was brightest for scoring distance from the periphery^95^. Bars indicate the average and dots represent mean % from individual experiments. N = 3. foci(embryos): WT = 699(50), *met-2Δ* = 869(57), *met-2-CD* = 850(59), *met-2Δ set-25Δ* = 819(51), *met-2-CD set-25Δ* = 820(56), (**d**) Representative Western blots and quantification measuring mCherry signal in WT (untagged), MET-2::mCherry, and MET-2-CD::mCherry). N = 3. ns = not significant by two-sided t-test. (**e**) iBAQ values showing enrichment of MET-2, ARLE-14, and LIN-65 from immunoprecipitation-MS/MS using LIN-65::GFP as bait in WT or *met-2-CD* embryos. Bars indicate the mean and dots represent individual measurements. Source data

To test directly whether MET-2 has a function beyond ensuring H3K9me deposition, we used CRISPR-mediated mutagenesis to compromise the active site of MET-2’s SET domain. The C1237A mutation *met-2(gw1660)* (hereafter *met-2-CD*) alters a highly conserved motif that is essential for the catalytic activity of SET domain proteins generally^45–47^ (Fig. 2a), and specifically of SETDB1 (ref. ^48^). Consistently, we find that H3K9me2 and H3K9me3 levels dropped to the same level in *met-2-CD* as in *met-2Δ* (Fig. 2b–d and Extended Data Fig. 2a,b). Moreover, H3K9me2 levels were as low in *met-2Δ set-25Δ* as in *met-2-CD set-25Δ* double mutants (Fig. 2c). Mass spectrometry suggested that H3K9me1 might not be completely abolished in the *met-2-CD* strain (Extended Data Fig. 2b), although this potential difference in H3K9me1 could not be detected by western blot (Extended Data Fig. 2a), nor was *met-2-CD* sufficient to preserve H3K9me1/2/3-dependent perinuclear anchoring of a well-characterized heterochromatic reporter (Extended Data Fig. 2c)^7,49^. Taken together, we conclude that *met-2-CD* is catalytically deficient for H3K9 methylation yet does not compromise MET-2 protein levels (Extended Data Fig. 2d) or MET-2’s ability to bind ARLE-14 or LIN-65 (Extended Data Fig. 2e). This mutant made it possible to monitor H3K9me2-independent functions of MET-2.

We introduced the *met-2-CD* mutation (C1237A) into the *met-2::mCherry* locus to track catalytically deficient MET-2 in live embryos. We found that MET-2-CD forms foci, and the number of foci did not differ from WT MET-2 (Fig. 2e). Both WT and MET-2-CD foci were dependent on LIN-65, and thus we concluded that MET-2 foci can form independently of HMT catalytic activity. This allowed us to distinguish the physiological effects arising from a loss of H3K9me2 from those caused by the dispersion of MET-2 foci.

Because fertility is sensitive to the loss of H3K9me, we next scored progeny in *met-2-CD* animals. Despite being H3K9me-deficient, the *met-2-CD* population retained higher levels of fertility than *met-2Δ* animals, and this was independent of *set-25* (Fig. 2f). Previous work has shown that *met-2Δ* animals also display a stochastic delay in development^30^, which we quantified by scoring the number of L1 larvae that develop to the L4 larval stage by 42 h in a synchronized culture at 20 °C. In contrast to *met-2Δ*, *met-2-CD* animals had no notable developmental delay (Fig. 2g). Moreover, the rescue of development timing was reduced upon *lin-65* ablation (Fig. 2g). In conclusion, a LIN-65-dependent function of MET-2-CD is important for normal development in both somatic and germline tissues, suggesting a role for MET-2 foci that is independent of MET-2’s HMT activity.

### MET-2-CD can repress heterochromatic genes

**Fig. 3:**
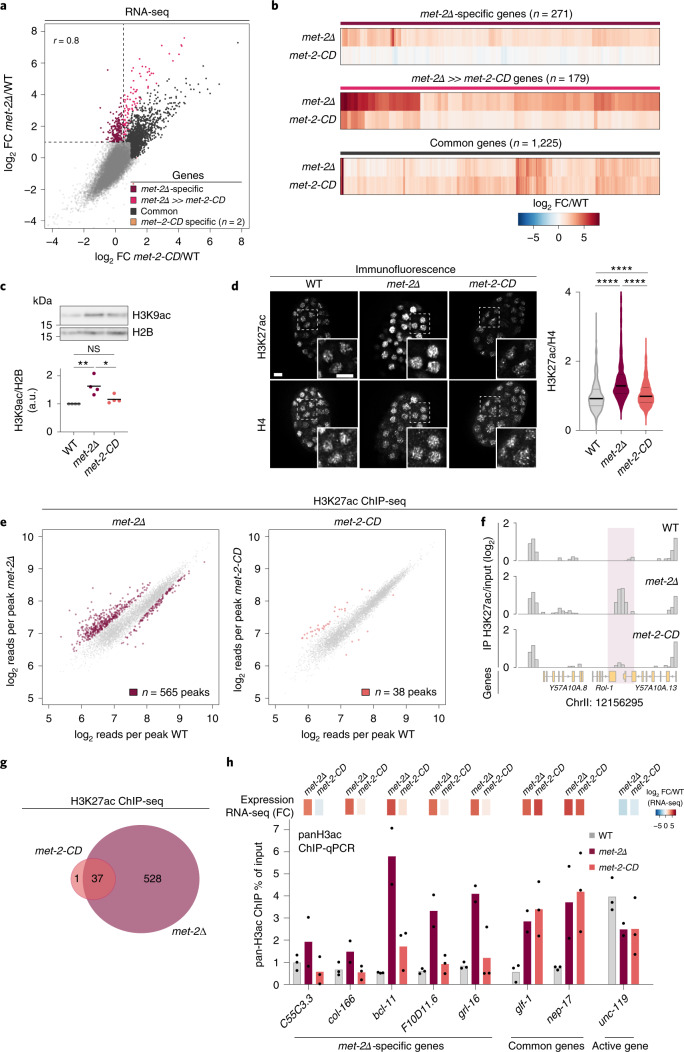
MET-2-CD foci mitigate transcriptional activation and histone acetylation. **a**, Correlation of RNA-seq log_2_ FCs between *met-2Δ* and *met-2-CD* relative to WT embryos. Genes significantly upregulated to WT in both *met-2Δ* and *met-2-CD* are colored in black (FDR < 0.01; FC > 2). Genes that are upregulated to WT and >2-fold higher in the *met-2Δ* compared with *met-2-CD* are colored in red (FDR < 0.01). Dotted line demarcates *met-2Δ*-specific genes (FDR < 0.01 and FC > 2 in *met-2Δ/WT*, and FC < 0.1 in *met-2-CD/WT*). Pearson correlation coefficient (*r*) is displayed. *N* = 3. **b**, log_2_FC of *met-2Δ*-specific genes, *met-2Δ»met-2-CD* and common genes in *met-2Δ* and *met-2-CD*. **c**, Representative western blot and quantification comparing H3K9ac levels in WT, *met-2Δ* and *met-2-CD* embryos. *N* = 4. *P*(*met-2Δ*, *met-2-CD*) = 0.038, *P*(WT, *met-2Δ*) = 0.0078. **d**, Immunofluorescence and quantification of mean H3K27ac signal per nucleus normalized to H4 in WT, *met-2Δ* and *met-2-CD*. Scale bar, 5 µm. *N* = 3, *n* nuclei(embryos): WT = 780(44), *met-2Δ* = 778(47), *met-2-CD* = 765(38). Median and quartiles shown. *P*(WT, *met-2Δ*) < 2.0 × 10^−16^, *P*(WT, *met-2-CD*) = 1.1 × 10^−5^, *P*(*met-2Δ, met-2-CD*) < 2.0 × 10^−16^ by two-sided Wilcoxon signed-rank test. **e**, H3K27ac ChIP-seq in *met-2Δ* or *met-2-CD* early embryos compared with WT. Scatterplot correlates the mean number of reads per peak (normalized to input, log_2_) in *met-2Δ* or *met-2-CD* with WT. Differentially enriched peaks for each genotype versus WT are highlighted in color (FDR < 0.01). *N* = 3. **f**, H3K27ac ChIP-seq exemplar region of Chr II is shown (*N* = 3). Pink box highlights a peak with gain of acetylation in *met-2Δ* embryos but not in *met-2-CD*. **g**, Venn diagram of H3K27ac differentially enriched peaks in ChIP-seq strains shown in **e** compared with WT (FDR < 0.01). **h**, Expression heatmap and pan-H3ac ChIP-qPCR at representative *met-2Δ*-specific genes, common genes or the *met-2* independent gene *unc-119*. Bars indicate the mean and dots represent individual measurements. *N* = 3 (*met-2Δ* *N* = 2). ChIP-qPCR, Chromatin Immunoprecipitation followed by quantitative real-time PCR; *P < 0.01, **P < 0.001, ****P < 0.00001. Source data

It was unclear whether MET-2-CD would be sufficient to preserve gene repression, despite the drop in H3K9me2 found in this mutant. We performed transcriptome analysis in *met-2-CD*, *met-2Δ* and WT early embryos. Importantly, most of the genes upregulated in *met-2Δ* were also upregulated in *met-2-CD*, highlighting the importance of H3K9me in maintaining repression (Fig. 3a, ‘common’ genes, *N* = 1225). However, there were 271 *met-2Δ* targets that were upregulated in *met-2Δ* but not in *met-2-CD* (‘*met-2Δ*-specific’ genes) and 179 genes that exhibited a mitigated response (derepression was more than 2-fold lower in *met-2-CD* than in *met-2Δ*, ‘*met-2Δ* ≫ *met-2-CD*’, Fig. 3b). In other words, 28% of *met-2* target genes retained complete or partial repression in *met-2-CD* embryos.

**Extended Data Fig. 3:**
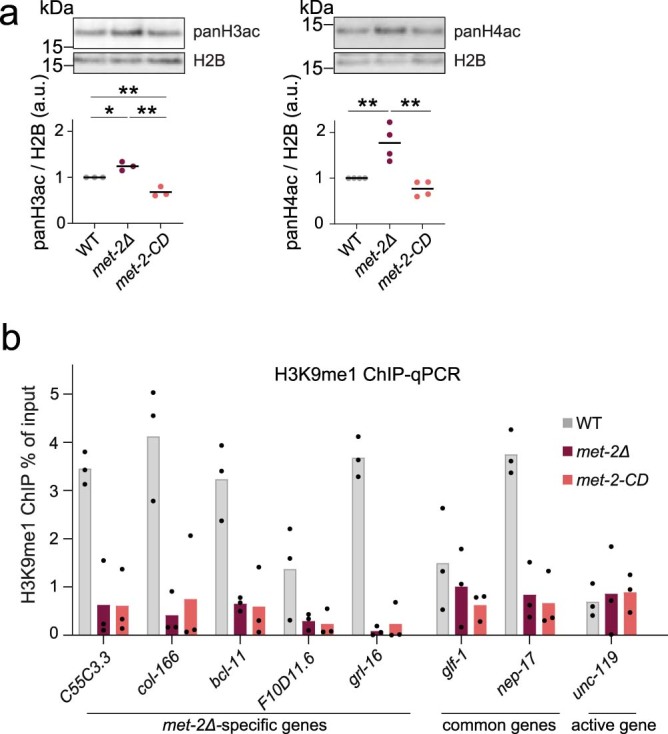
MET-2-CD inhibits acetylation independent of H3K9me. (**a**) Representative Western blots and quantification comparing WT, *met-2Δ*, and *met-2-CD* embryo acetylation levels. panH3ac N = 3, p(WT, *met-2Δ*) = 0.011, p(WT, *met-2-CD*) = 6.4*10^-3^, p(*met-2Δ*, *met-2-CD*) = 3.2*10^-3^; panH4ac N = 4, p(WT, *met-2Δ*) = 7.4*10^-3^, p(*met-2Δ*, *met-2-CD*) = 2.3*10^-3^ by two-sided t-test. (**b**) H3K9me1 ChIP-qPCR at representative *met-2Δ*-specific genes, common genes, or the *met-2* independent gene *unc-119*. Bars indicate the mean and dots represent individual measurements. N = 3. Source data

Heterochromatin is not only marked by H3K9me but is also characterized by a general hypoacetylation of histone tails. In contrast, gene activation is associated with increased histone tail acetylation. Intriguingly, mass spectrometry showed a global increase in H3K9 acetylation in *met-2Δ* embryos that was absent in *met-2-CD* embryos (Extended Data Fig. 2b). We confirmed this result by western blot and immunofluorescence, showing that both histone H3 and H4 acetylation (Extended Data Fig. 3a), including H3K9ac and H3K27ac (Fig. 3c,d), were increased in *met-2Δ* embryos, but not in *met-2-CD* embryos. These results suggested that the aberrant gain of acetylation in *met-2Δ* animals is not an inevitable consequence of H3K9me loss.

To see which loci gain histone acetylation in *met-2* mutants, we performed Chromatin immunoprecipitation followed by genome-wide sequencing (ChIP-seq) in early embryos. We concentrated on H3K27ac due to its well-described role in transcriptional activation and enhancer function^50^. Relative to WT embryos, we defined 565 differentially enriched H3K27ac peaks in *met-2Δ*, while only 38 changes were detected in *met-2-CD* (Fig. 3e,f). All but one of the rare *met-2-CD* peaks were also observed in *met-2Δ* embryos (Fig. 3g). In addition, we examined the enrichment for pan-histone H3 acetylation (H3ac) at a subset of *met-2Δ*-specific genes by Chromatin Immunoprecipitation followed by quantitative real-time PCR (ChIP-qPCR). Compared with WT, these loci were enriched for H3ac in *met-2Δ* but not in *met-2-CD* embryos (Fig. 3h), whereas for common targets or a nonrepressed control, H3ac enrichment was very similar in *met-2Δ* and *met-2-CD* strains (Fig. 3h). The lack of acetylation in the *met-2-CD* embryos was not linked to residual H3K9me1, as the two mutants (*met-2-CD* and *met-2Δ*) had equally low levels of H3K9me1 at these loci (Extended Data Fig. 3b). Instead, the data suggested that MET-2 contributes to transcriptional repression and blocks the acetylation of specific loci, independent of its HMT activity.

### ARLE-14 promotes MET-2 association with chromatin

**Fig. 4:**
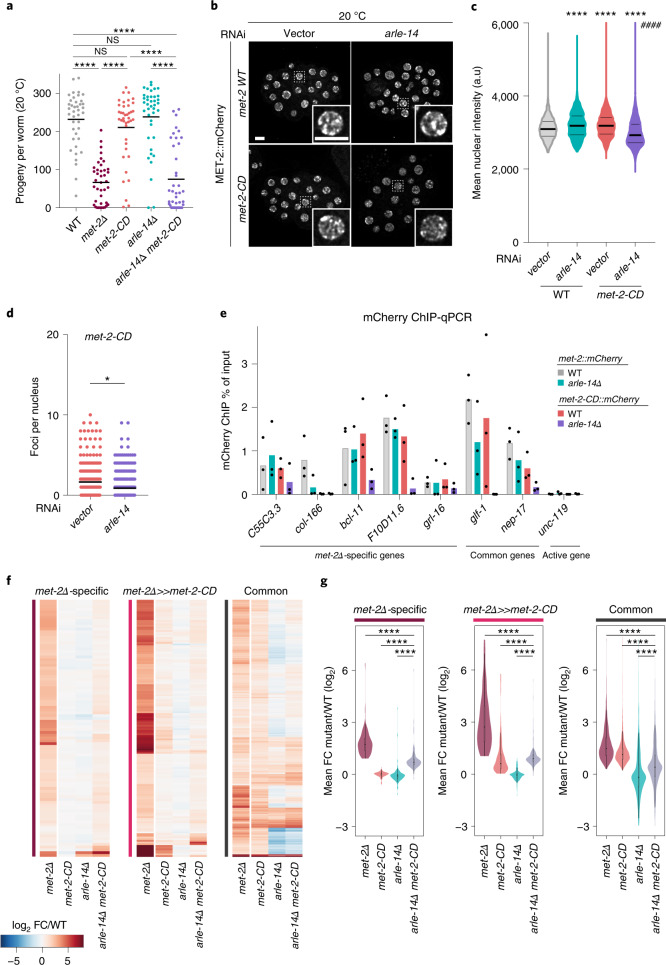
ARLE-14 stabilizes MET-2 at chromatin redundantly with H3K9me. **a**, Brood sizes of the indicated strains at 20 °C. *N* = 2, *n* = 40. *P*(WT, *met-2Δ*) < 2.0 × 10^−16^, *P*(WT, *met-2-CD*) = not significant, *P*(WT, *arle-14Δ*) = not significant, *P*(WT, *arle-14Δ met-2-CD*) = 1.94 × 10^−12^, *P*(*met-2Δ, met-2-CD*) = 8.2 × 10^−14^, by one-way ANOVA, followed by Tukey post hoc test. **b**–**d**, Live imaging of expressing either WT MET-2::mCherry or MET-2-CD::mCherry ± *arle-14* RNAi (**b**), and quantification of MET-2 nuclear signal *P*(WT(*vector*), WT(*arle-14*)) = 1.0 × 10^−12^, *P*(WT(*vector*), *met-2-CD*(*vector*)) < 2.0 × 10^−16^, *P*(WT(*vector*), *met-2-CD*(*arle-14*)) = 5.7 × 10^−7^, *P*(*met-2-CD*(*vector*), *met-2-CD*(*arle-14*)) < 2.0 × 10^−16^ (**c**) and MET-2 foci per nucleus (**d**). *P*(*met-2-CD*(vector), *met-2-CD*(*arle-14* RNAi)) = 0.041. Scale bar, 5 µm. *N* = 3, *n* = 45 embryos. Nuclei: *met-2::mCherry*: vector = 816, *arle-14* RNAi = 1,199, *met-2-CD::mCherry*: vector = 1,056, *arle-14* RNAi = 1,787. *P* values calculated by two-sided Wilcoxon signed-rank test. **e**, mCherry ChIP-qPCR at representative *met-2Δ*-specific genes, common genes or the *met-2*-independent gene *unc-119*. Bars indicate the mean and dots represent individual measurements. *N* = 3. Statistical comparison of *arle-14Δ met-2-CD* with *met-2-CD*: *C55C3.3* *P* = 0.05, *col-166* *P* = 0.74, *bcl-11* *P* = 0.004, *F10D11.6* *P* = 0.009, *grl-16* *P* = 0.34, *glf-1* *P* = 0.0024, *nep-17* *P* = 0.05, *unc-119* *P* = 0.15, Student’s *t*-test. **f,** heatmap of log_2_ FC of *met-2Δ*-specific genes, *met-2Δ»met-2-CD* genes and common genes in *met-2Δ*, *met-2-CD, arle-14Δ* and *arle-14Δ met-2-CD* relative to WT. **g**, distribution of log_2_ FC of the same comparisons as in (**f**) displayed as violin plots*.* *met-2Δ*-specific: *P*(*met-2Δ*, *arle-14Δ met-2-CD*) < 2.0 × 10^−16^, *P*(*met-2-CD*, *arle-14Δ met-2-CD*) < 2.0 × 10^−16^, *P*(*arle-14Δ*, *arle-14Δ met-2-CD*) < 2.0 × 10^−16^; *met-2Δ*»*met-2-CD*: *P*(*met-2Δ*, *arle-14Δ met-2-CD*) < 2.0 × 10^−16^, *P*(*met-2-CD*, *arle-14Δ met-2-CD*) = 0.00012, *P*(*arle-14Δ*, *arle-14Δ met-2-CD*) < 2.0 × 10^−16^; common: *P*(*met-2Δ*, *arle-14Δ met-2-CD*) < 2.0 × 10^−16^, *P*(*met-2-CD*, *arle-14Δ met-2-CD*) < 2.0 × 10^−16^, *P*(*arle-14Δ*, *arle-14Δ met-2-CD*) < 2.0 × 10^−16^ by two-sided Wilcox rank sum test. *N* = 3. Boxplots show median, boxes 50% and whiskers 90% of each group. NS, not significant; *P < 0.01, **** and #### *P* < 0.00001. Source data

**Extended Data Fig. 4:**
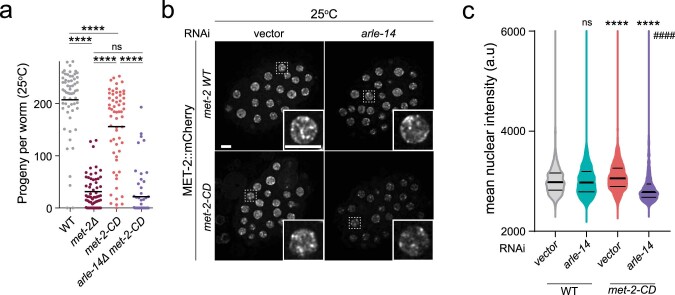
ARLE-14 is required for fertility and nuclear enrichment of MET-2 in *met-2-CD* animals. (**a**) Brood sizes of the indicated strains at 25 °C. N = 3, n = 60. ns = not significant, p(WT,met-2Δ)< 2.0*10^-16^, p(WT,met-2-CD) = 4.2*10^-6^, p(met-2Δ,met-2-CD) < 2.0*10^-16^, p(met-2Δ, arle-14Δ met-2-CD) = 0.77,by one-way ANOVA, followed by Tukey post-hoc test. (**b**-**c**) Live imaging of embryos expressing either WT MET-2::mCherry or MET-2-CD::mCherry + /- *arle-14* RNAi grown at 25 °C and (c) quantification of MET-2 nuclear signal. p(WT(*vector*), WT(*arle-14*))=not significant, p(WT(*vector*), *met-2-CD*(*vector*))=1.1*10^-9^, p(WT(*vector*),*met-2-CD(arle-14)*) < 2.0*10^-16^, p(*met-2-CD*(*vector*),*met-2-CD(arle-14)*)<2.0*10^-16^ Low nuclear mCherry signal precluded foci quantification. Scale bar = 5 µm. N = 3 N = 3, n = 45 embryos. nuclei: *met-2::mCherry*: vector = 884, *arle-14* RNAi = 1823, *met-2-CD::mCherry*: vector = 1076, *arle-14* RNAi = 878. ns = not significant, ****p-adj < 10^-4^ compared to WT vector, ^####^p-adj < 10^-4^ compared to MET-2-CD vector by two-sided Wilcoxon signed-rank test. Source data

After determining that catalytic-dependent and -independent roles for MET-2 exist in gene repression, we sought to see if ARLE-14 plays a role in the noncatalytic MET-2 function. If ARLE-14 simply promotes MET-2 HMT activity, *met-2-CD* would be epistatic with *arle-14*. We examined fertility at both 20 °C and 25 °C in *arle-14Δ met-2-CD* double mutant animals. Remarkably, at 20 °C neither *arle-14Δ* nor *met-2-CD* single mutants had significantly fewer progeny than WT animals (Fig. 4a). However, the *arle-14Δ met-2-CD* population showed a synthetic loss of viable offspring at both temperatures, phenocopying *met-2Δ* mutants (Fig. 4a and Extended Data Fig. 4a). This was surprising, because *arle-14Δ* did not affect foci of WT MET-2 (Figs. 1c and 2e). Nonetheless, we found that *arle-14* was required both for MET-2-CD::mCherry foci number and the overall nuclear abundance of the HMT (Fig. 4b–d), an effect that was enhanced at 25 °C (Extended Data Fig. 4b,c). These data argued that ARLE-14 acts redundantly with H3K9me to stabilize MET-2 in foci.

Although the function of worm ARLE-14 is unclear, recent evidence in human cells suggested that the human homolog, ARL14EP, may facilitate SETDB1 interaction with chromatin^43^. To see if ARLE-14 promotes MET-2 binding to chromatin, we performed ChIP-qPCR using antibodies against MET-2::mCherry in both WT and *met-2-CD* backgrounds, both in the presence and absence of ARLE-14. MET-2-CD showed similar enrichment as WT MET-2 at five of seven tested targets (Fig. 4e). Loss of *arle-14* showed little effect on WT MET-2::mCherry enrichment at most MET-2 gene targets (with the exception of *col-166*), but *arle-14* did lead to the loss of MET-2-CD at these same sites (Fig. 4e). Consistently, the loss of ARLE-14 in *met-2-CD* embryos led to the upregulation of *met-2Δ*-specific targets based on RNA-seq data, which was not observed in *arle-14*Δ alone (Fig. 4f,g). Taken together, these data suggested that ARLE-14 acts redundantly with H3K9me to stabilize both MET-2 foci and its association with MET-2-controlled genes. Thus, *arle-14* is needed for the noncatalytic function of MET-2 on both physiological and molecular levels.

### Acute heat stress dissociates MET-2 foci

**Fig. 5:**
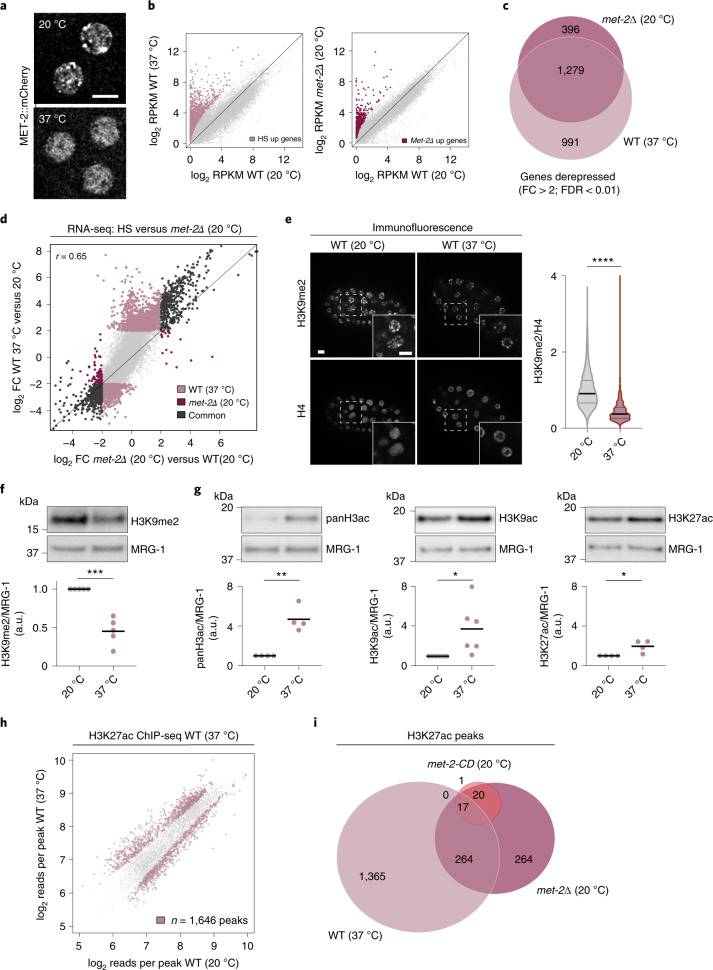
Heat stress destabilizes MET-2 foci and heterochromatin. **a**, Representative images of MET-2::mCherry foci in live WT embryos cultured normally (20 °C) or heat-stressed (HS; 37 °C, 60 min). Scale bar, 5 µm. *N* = 3. **b**, RNA-seq comparing the gene expression in RPKM (reads per kilobase million) of HS treatment or *met-2Δ* relative to WT(20 °C) early embryos. Genes marked in light purple and dark red are upregulated in 37 °C-treated embryos and *met-2Δ*, respectively, relative to WT(20 °C) (FDR < 0.01; log_2_FC > 2). *N* = 3. **c**,**d**, Venn diagram of derepressed genes (**c**) and correlation between HS-treated and *met-2Δ* log_2_FC in gene expression compared with WT(20 °C) (**d**). WT 37 °C versus *met-2Δ*: *P* < 2.2 × 10^−16^ by two-sided Fisher’s exact test. Pearson correlation coefficient (*r*) is displayed. **e**, Immunofluorescence and quantification of H3K9me2 per nucleus normalized to H4 in WT(20 °C) and 30-min HS-treated embryos. Scale bar, 5 µm. *N* = 3, *n* nuclei(embryos): 20 °C = 1,522(51), 37 °C = 1,139(46). Median and quartiles shown. *P* < 2.0 × 10^−16^ by two-sided Wilcoxon signed-rank test. **f**,**g**, Representative western blots and quantification of H3K9me2 (**f**) or H3ac (**g**) normalized to MRG-1 in WT(20 °C) and HS-treated WT embryos. HS: 37 °C, 30 min (**f**) or 60 min (**g**). H3K9me2 *N* = 5, *P* = 1.1 × 10^−4^; pan-H3ac *N* = 4, *P* = 0.0012; H3K9ac *N* = 5, *P* = 0.025; H3K27ac *N* = 4, *P* = 0.018 by two-sided *t*-test. **h**, H3K27ac ChIP-seq HS-treated early embryos compared with WT(20 °C). Differentially enriched peaks are highlighted in color (FDR < 0.01). *N* = 3. **i**, Venn diagram of H3K27ac differentially enriched peaks in HS treatment versus *met-2* mutants shown in Fig. 5h and Fig. 3e relative to WT (20 °C) (FDR < 0.01). WT 37 °C versus *met-2Δ*: *P* < 2.2 × 10^−16^ by two-sided Fisher’s exact test. *P < 0.01, **P < 0.001, ***P < 0.0001, ****P < 0.00001. Source data

**Extended Data Fig. 5:**
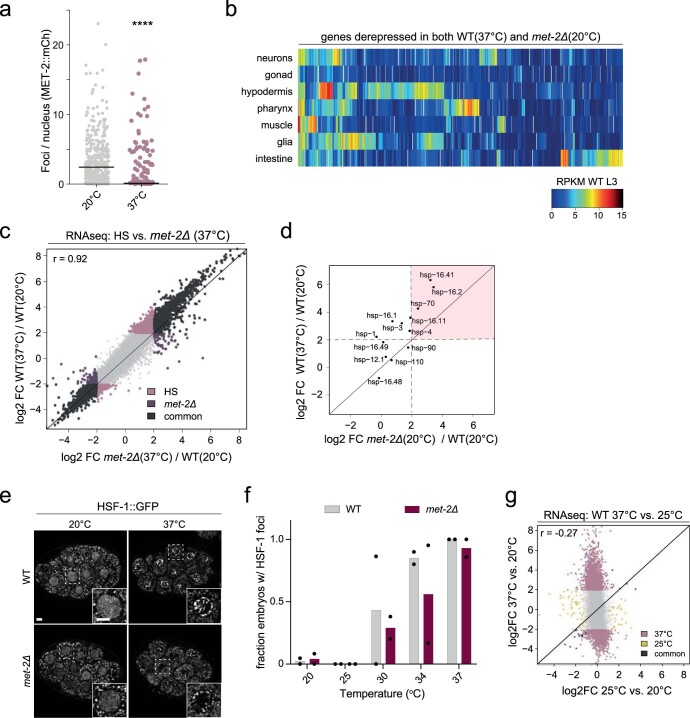
Loss of *met-2* or heat stress activates transcription in an HSF-1 independent manner. (**a**) Quantification of foci loss shown in Fig. 5a, from ref. ^14^. ****p-adj=1.5*10^-9^ by two-sided Wilcoxon signed-rank test. (**b**) Genes de-repressed in both *met-2Δ* mutant and WT(37 °C) early embryos (Fig. 5d, black circles) are normally expressed in differentiated tissues^92^. Heatmap shows the expression (RPKM) in WT L3 larvae of each derepressed gene (columns) in individual tissues (rows). (**c**) Correlation of gene expression in WT and *met-2Δ* embryos both treated with HS (37 °C, 1 hr) relative to WT(20 °C). Pearson correlation coefficient (r) is displayed. (**d**) Expression changes of all genes designated Heat Shock Protein (*hsp*) in WT(37 °C) and *met-2Δ* relative to WT(20 °C). Pink box = log2 FC > 2 in both WT-HS and *met-2Δ*−20 °C. (**e**) Representative images of HSF-1::GFP protein in live WT or *met-2Δ* embryos + /- HS. HS: 37 °C, 30 min. Scale bar = 5 µm. (**f**) Fraction of embryos exhibiting HSF-1::GFP foci after 30 min at the indicated temperature. N = 2, n embryos = 20-60 per temperature. (**g**) Correlation of expression between embryos treated with HS (37 °C, 1 h) and embryos from parents grown at 25 °C^6^, both compared to WT(20 °C). N = 3. Source data

Having shown by genetics that MET-2 has a noncatalytic function that correlates with its concentration into foci, we wondered whether foci dynamics might be physiologically relevant in animals containing normal levels of H3K9me. We previously showed that MET-2 foci disperse upon transient exposure to 37 °C heat shock (HS) (Fig. 5a and Extended Data Fig. 5a)^14^, coincident with the canonical heat shock response (HSR)^51^. Intriguingly, although the HSR is clearly associated with well-described signaling pathways that dampen transcription while specifically upregulating protein chaperone genes^52,53^, earlier studies in other species also linked acute heat stress to the transcriptional derepression of silent chromatin^54–58^. Thus, HS conditions provided a physiological system in which we could monitor a transcriptional response that coincides with the induced dispersion of MET-2 foci, without genetic alteration.

We performed RNA-seq on early embryos exposed to either normal temperature (20 °C) or HS (1 h at 37 °C). We observed a broad upregulation of genes at 37 °C relative to embryos kept at 20 °C (2026 genes up, 314 genes down, fold change (FC) > 4, false discovery rate (FDR) < 0.01, Fig. 5b). Consistent with the dispersion of MET-2 foci, we found that most HS-upregulated genes, similar to *met-2Δ*-upregulated genes, had very low expression levels in embryos cultured at 20 °C (80% reads per kilobase million < 4 at 20 °C Fig. 5b). Strikingly, we observed a positive correlation (*r* = 0.65) between the transcriptional changes induced by 37 °C versus those induced by the loss of *met-2* at 20 °C versus WT (20 °C) embryos (Fig. 5b–d). In fact, 76% of the *met-2Δ* target genes were upregulated under HS conditions, constituting 56% of all HS-derepressed genes (Fig. 5c). The majority of the shared targets were genes that are normally expressed in postembryonic, differentiated tissues (Extended Data Fig. 5b). These data suggest that embryos exposed to HS not only lose MET-2 foci but also derepress many genes that are normally repressed by MET-2 (Fig. 5c,d). On the other hand, *met-2Δ* embryos respond to HS similarly to WT animals with respect to global gene expression, HSP gene induction and HSF-1 foci formation (Extended Data Fig. 5c–f).

Because *met-2Δ* mutants present enhanced phenotypes at 25 °C (refs. ^6,30^), we wondered whether the shift from 20 °C to 25 °C also induces a similar stress response as exposure to 37 °C. However, no HSF-1 foci formed in either WT or *met-2Δ* embryos at 25 °C, nor did the transcriptome of animals grown at 25 °C correlate with the pattern of genes induced by HS (Extended Data Fig. 5f,g). Thus, the loss of fertility at 25 °C is not a heat shock response.

**Extended Data Fig. 6:**
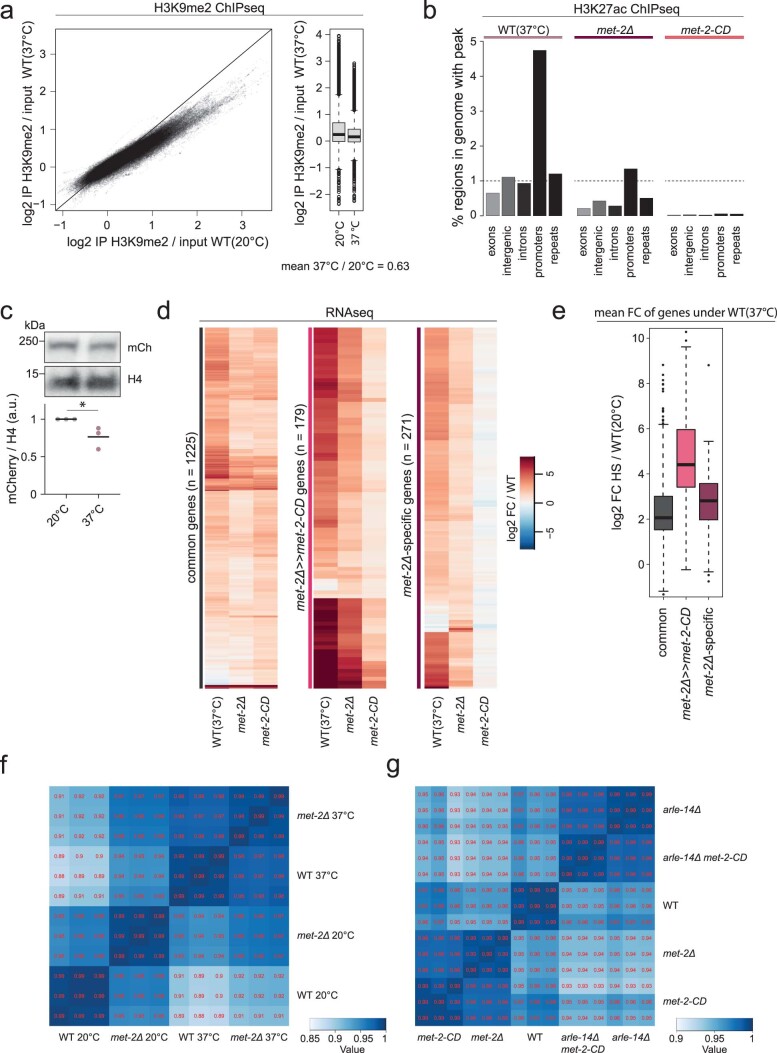
Heat stress leads to permissive transcriptional state. (**a**) Comparison of H3K9me2 genome wide enrichment (500 bp tiles) over input (log2) between HS (37 °C, 60 min) and WT(20 °C) embryos via ChIP-seq (N = 2). Boxplots show median, boxes 50% and whiskers 90% of each group. Correlation between replicates: HS = 0.97, WT(20 °C) = 0.98. (**b**) Percentage of defined genomic elements that overlap with peaks differentially enriched for H3K27ac in HS treatment (37 °C, 60 min), *met-2Δ*, and *met-2-CD* embryos relative to WT(20 °C). (**c**) Representative Western blot and quantification of MET-2::mcherry in embryos + /- HS: 37 °C, 60 min. N = 3. *p = 0.050 by two-sided t-test. (**d**-**e**) Log2 FC of *met-2Δ*-specific genes, *met-2Δ»met-2-CD*, and common genes in HS (37 °C, 60 min), *met-2Δ* and *met-2-CD* and (d) mean fold change observed in HS in each gene set. N = 3. Boxplots show median, boxes 50% and whiskers 90% of each group. (**f**-**g**) Correlations between RNAseq replicas for (e) WT and *met-2Δ* + /- HS (37 °C, 60 min), and (f) WT, *met-2Δ*, *met-2-CD*, *arle-14Δ*, and *arle-14Δ met-2-CD*. Source data

Given that the loss of MET-2 foci correlates with a loss of H3K9me (Extended Data Fig. 1b), we examined how H3K9me levels change during 30 min at 37 °C. Indeed, embryos exposed to HS displayed a drop in H3K9me2 to 45% or 50% of untreated levels, as measured by western blot or immunofluorescence, respectively (Fig. 5e,f). Whereas the *met-2* messenger RNA level upon HS was unchanged (log_2_FC = −0.094), we scored a ~24% reduction in MET-2 protein level (Extended Data Fig. 6c), which may influence chromatin states. We examined regions that bear H3K9me2 in embryos at 20 °C by ChIP-seq, to see if H3K9me2 is also reduced in a localized manner after exposure to 37 °C for 1 h. Indeed, we observed a general reduction in read counts for H3K9me2 to 63% of level scored at 20 °C (Extended Data Fig. 6a).

Whereas the transcriptional induction following HS was stronger than what we observed in *met-2Δ*, the loss of H3K9me2 was less. Given our earlier data that link the loss of MET-2 foci with increased acetylation levels, we checked to see if histone acetylation changes during HS. Western blots showed that HS-treated embryos have a substantial increase in pan-H3ac, including increases in H3K9ac and, more modestly, H3K27ac (Fig. 5g). Taken together, our results argue that HS-induced dispersion of MET-2 foci coincides with a partial destabilization of H3K9me2, a strong gain of H3ac and widespread transcriptional activation of loci silenced by MET-2 under normal conditions.

To compare the changes during HS to those incurred by *met-2Δ*, we examined changes in H3K27ac in WT embryos (HS versus 20 °C) by ChIP-seq, and compared these peaks with data from our *met-2Δ* and *met-2-CD* animals. HS (30 min at 37 °C) led to an enrichment of H3K27ac on 1646 peaks (Fig. 5h). Importantly, 49.7% of the H3K27ac peaks in *met-2Δ* were shared with those in HS embryos (Fig. 5i). Nonetheless, there were three times as many peaks after HS as in unstressed *met-2Δ* embryos (1646 in HS versus 565 in *met-2*), consistent with the idea that HS mobilizes a broad transcriptional response, as well as destabilizing MET-2 foci. Other regions of the genome of HS-treated embryos actually lost H3K27ac, reflecting the fact that a certain degree of transcriptional downregulation occurs during the canonical HSR^59^. The sites that gained acetylation in both HS and *met-2Δ* embryos were mainly promoters and repeats, while *met-2-CD* embryos showed almost no gain in acetylation over WT (Extended Data Fig. 6b). Intriguingly, the *met-2* target genes that remain fully or partially repressed in the *met-2-CD* background (presumably because they remain in foci) showed particularly pronounced derepression during HS-induced loss of focus formation (Extended Data Fig. 6d,e). In other words, the dissolution of MET-2 foci upon HS is coupled with increased acetylation and gene derepression, beyond the program induced by HSF-1, reinforcing the correlation we found between MET-2-CD foci and transcriptional repression.

## Discussion

**Fig. 6:**
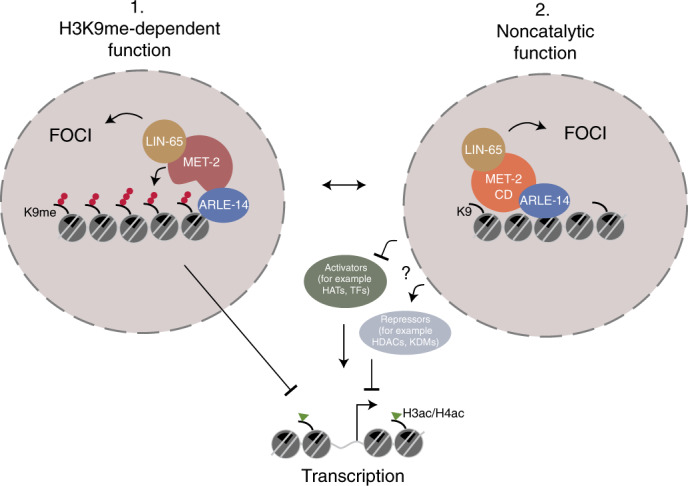
The deposition of H3K9 methylation acts in parallel with a proposed structural role of MET-2 in transcriptional repression. The primary function of the SETDB1-like enzyme, MET-2, is to mediate H3K9 methylation (1). Its loss leads to extensive gene and repeat derepression. The concentration of MET-2, LIN-65 and ARLE-14 into nuclear foci requires LIN-65 and represses transcription by H3K9me-independent inhibition of histone acetylation in parallel to ensuring efficient H3K9 methylation (2). ARLE-14 stabilizes MET-2 foci at chromatin redundantly with H3K9me. HATs, histone acetyl transferases; KDMs, histone demethylases; TFs, transcription factors.

The sequestration and self-association of heterochromatin into foci has been observed since the early days of microscopy^1^. While these foci coincided with HP1 and with H3K9me-specific HMTs, it was unclear whether the localized concentration of these proteins simply enhanced the propagation of H3K9 methylation, or served another role. Using a mutant of a key residue in the SET domain of the SETDB1-like enzyme MET-2 (*met-2-CD)*, we have been able to distinguish a repressive role for MET-2 focus formation distinct from the catalytic function of MET-2’s SET domain. H3K9me is clearly the key regulatory mark for the stable silencing of lineage-specific genes and repetitive elements across animal species, and the majority of loci repressed by heterochromatin in worms depend on the activity of MET-2’s SET domain. Yet the careful analysis of the *met-2-CD* allele has revealed a second, noncatalytic role for MET-2 that requires its binding partners LIN-65 (*H.s*. ATF7IP) and ARLE-14 (*H.s*. ARL14EP), and which correlates with the accumulation of MET-2 and its ligands in subnuclear foci (Fig. 6).

LIN-65 is necessary for focus formation, while ARLE-14 may stabilize MET-2 interaction with chromatin within foci, acting redundantly with H3K9me with respect to MET-2 recruitment and focus formation (ref. ^14^) (Fig. 6). Foci of MET-2-CD appear to be sufficient to block local acetylation and mitigate inappropriate transcription of selected developmentally regulated genes, in an ARLE-14-dependent manner. We infer that MET-2 foci not only correlate with efficient H3K9me, but they create a semirepressed state that is refractory to histone acetylation. Consistent with the repressive function, MET-2-CD was able to largely preserve fertility and somatic development in the absence of MET-2’s catalytic activity under normal growth conditions (20–25 °C). For this function, however, it requires ARLE-14.

The comparison of the *met-2Δ* and *met-2-CD* transcriptomes showed that even among the loci that were derepressed in both *met-2Δ* and *met-2-CD* embryos, the transcriptional levels of many genes were higher in *met-2Δ*. Genes that retained full or partial repression constitute 28% of *met-2* target genes. This argues that H3K9me and MET-2’s noncatalytic function silence genes in a partially additive manner, reinforcing each other to maintain heterochromatic repression.

The mechanism of focus-mediated repression appears to be largely structural: we found that MET-2-CD foci were able to block the increase in histone tail acetylation found in *met-2Δ* mutants. We note that similar to H3K9me, histone hypoacetylation is a hallmark of heterochromatin across eukaryotic species^60,61^. In *Schizosaccharomyces pombe* the histone deacetylases (HDACs) Clr3 and Sir2 are essential to restrict RNA Pol II access to heterochromatin^62,63^, and the treatment of murine cells with HDAC inhibitors resulted in a widespread loss of heterochromatic repression^64^ and impaired the establishment of lineage-specific expression patterns in differentiating mouse embryos^65^. Finally, histone acetylation itself is known to promote chromatin decompaction, weaken the interaction between histones and DNA, and antagonize phase separation of chromatin arrays in vitro^66–69^. Therefore, MET-2-CD’s ability to prevent hyperacetylation could explain how it maintains the semirepressed state that we observe by RNA-seq. MET-2-CD may block acetyltransferase access directly or recruit the factors, such as HDACs, that ensure that heterochromatin remains silent.

We cannot yet determine whether MET-2/LIN-65/ARLE-14 preserve hypoacetylation directly or indirectly. Although no HDACs were enriched in our MET-2 pulldown^14^ (Fig. 1b), it was recently suggested that MET-2 can physically associate with HDA-1 (HDAC1) in a SUMOylation-dependent manner^70^. In addition to its physical interaction with LIN-65 and ARLE-14, and potentially HDAC1, MET-2 interacts genetically with the H3K4 demethylase SPR-5 (ref. ^71^), the DREAM complex^72^ and SMARC-1 chromatin remodelers^73^. While we do not exclude it, the hypothesis that these factors serve as effectors of MET-2’s noncatalytic function is made less likely by the fact that each of the others was shown to act either redundantly with MET-2 or through H3K9me. Alternatively, recent investigations into the function of subnuclear condensates suggest that these foci may physically impair the access of transcription activating complexes, such as RNA Pol II, Mediator, basal transcription factors or histone acetyltransferases^74^. Because foci of MET-2 and its cofactors share properties of condensates, they may also block recruitment of the transcription machinery and thereby inhibit the activation and acetylation of silenced genes, despite reduced levels of H3K9me.

We note that we were unsuccessful in expressing recombinant MET-2 or LIN-65, potentially due to their large size and highly disordered domains^75,76^. This precluded in vitro tests with recombinant protein for direct binding and/or HMT activity, which would complement the extensive genetic and biochemical evidence implicating MET-2 as the primary H3K9me1/me2 HMT in *C. elegans* embryos^7,15,28,30,32,71,77–82^. Nevertheless, we were able to demonstrate H3K9me activity of MET-2 recovered in a pull-down assay. We note that MET-2-CD has the same protein stability, localization, dynamics and protein interactions as WT MET-2, and yet H3K9me2 levels drop strikingly. This supports a direct role of MET-2 catalytic activity in H3K9me deposition, and a direct role for MET-2-CD in a secondary repressive function associated with LIN-65 dependent focus formation.

We find that ARLE-14 becomes essential to stabilize the MET-2-CD interaction with chromatin and its accumulation in foci, when catalytic function is compromised (Fig. 4). Consistently, the ARLE-14 homolog ARL14EP has been shown in vitro to bind H3 peptides in a SETDB1-independent manner^43^. We therefore propose that ARLE-14 helps recruit MET-2 to chromatin, acting independently of the H3K9me mark. It is possible that ARLE-14 has other roles in gene activation that are independent of MET-2, or else that ARLE-14 also prevents MET-2 mistargeting, given that some genes actually acquire, rather than lose, repression upon loss of *arle-14* (Fig. 4f).

Our study is not the first to suggest noncatalytic functions for a diverse group of HMTs. In mammalian cells, the H3K9 HMT G9a is able to promote repression by recruiting DNA methylation enzymes, independently of its catalytic activity^44,83^. Similarly, mammalian MLL3 and MLL4 do not require the enzymes’ H3K4 methylation activity to maintain enhancer activation in mice^84^. We speculate that in the case of MET-2, the noncatalytic function may allow for multiple layers of repressive control that respond to different stimuli and react with different kinetics, an example of which is the cellular response to heat stress (HS, 30 min to 1 h at 37 °C). HS resulted in hyperacetylation and activation of silenced genes, coincident with MET-2 focus dispersion, even though the loss of genome-wide H3K9me was less severe than that triggered by *arle-14Δ* or *met-2Δ* strains. The dissolution of MET-2 foci by HS illustrates a rapid transcriptional response that promotes chromatin opening while retaining H3K9me, potentially allowing a quick post-HS recovery of the prestress transcriptome.

In summary, we have uncoupled the effect of MET-2 concentration into subnuclear foci from its lysine methyltransferase activity. While H3K9me deposition is the primary driver of gene silencing, both H3K9me and MET-2 focus formation contribute to the repression of transcription and may mutually reinforce the heterochromatic state. Whereas MET-2 foci do not require the HP1-like factors HPL-1 and HPL-2 (ref. ^14^), they do depend on LIN-65, a protein rich in low-complexity sequence and disordered domains^14,18^. They are also stabilized by ARLE-14 in a manner redundant with H3K9 methylation, and they attenuate histone tail acetylation. This indicates that the SETDB1-like complex containing MET-2, ARLE-14 and LIN-65 contributes to gene repression beyond the targeted methylation of histone H3K9, with physiological consequences for organismal growth and fertility.

## Methods

### Strains and maintenance

Strains used in this study are listed in Supplementary Table 1. Unless otherwise indicated, experiments were performed using early embryos isolated from animals cultured at 20 °C. Embryos were isolated by standard bleaching procedures; embryos isolated from heat-stressed adults were isolated in buffers prewarmed to 37 °C. The conditions of HS are indicated for each experiment. RNA interference (RNAi) experiments were performed as previously described^14^. Briefly, synchronized L1s were fed HT115 bacteria expressing double stranded RNA (dsRNA) on nematode growth media supplemented with 1 mM IPTG and 100 µg ml^−1^ carbenicillin. Embryos isolated from these animals were analyzed as indicated. Synchronized animals fed either vector or *arle-14* RNAi were grown to L4 stage at 20 °C before being shifted to 25 °C overnight, and their progeny were imaged (Extended Data Fig. 4).

We used CRISPR as previously described^18,85,86^ to generate alleles *NLS*::*met-2*(*gw1786*), *arle-14*(*gw1584*) and *met-2*(*gw1660*). CRISPR RNA (crRNA) and homology directed repair (HDR) repair templates are listed in Supplementary Table 2. *arle-14*(*gw1584*) was later confirmed not to be a null allele by mass spectrometry.

### Live microscopy and immunofluorescence

Imaging was performed as previously described^14^. Briefly, all images were captured using Visiview software on a confocal spinning-disk microscope, the AxioImager M1 (Zeiss) with a Yokogawa CSU-X1 scanhead (Yokogawa), a Rolera Thunder camera (Photometrics) and an α plan-NEOFLUAR ×100/1.45 oil objective (Zeiss). Live embryos were mounted on 2% agarose pads in M9 buffer. For immunofluorescence, embryos were fixed for 5 min in 2% formaldehyde, then transferred to poly-l-lysine-coated slides and snap-frozen. Embryos were freeze-cracked and immediately treated with 100% ethanol precooled to −20 °C for 2 min then allowed to dry. After three 5-min washes using PBS with 0.25% Triton X-100 (PBS-X), slides were blocked in PBS-X + 2% milk for 1 h at room temperature and subsequently exposed to antibodies overnight at 4 °C in a humid chamber. After washing as above, slides were treated at room temperature with secondary antibodies diluted in PBS-X + 2% milk. Antibody dilutions are as follows: 1:500 mouse anti-H3K9me2 (MBL), 1:5,000 mouse anti-H3K27ac (gift from H. Kimura), 1:500 rabbit anti-H4 (Abcam), 1:1,000 goat anti-mouse Alexa Fluor 488 (A11001; Invitrogen) and 1:1,000 donkey anti-rabbit Alexa Fluor 555 (A31572; Invitrogen). After DAPI (1:2,000) staining in PBS-X for 10 min, and three washes, slides were mounted with ProLong Gold Antifade (Thermo Fisher Scientific). Images were deconvolved using the Huygens remote manager (http://www.huygens-rm.org/wp/). Nuclear mean intensity and number of foci were quantified using KNIME^87^. Nuclei were first identified using MET-2::mCherry fluorescence, then foci were detected with a Laplacian-of-Gaussian detector from TrackMate (fmi-ij2-plugins-0.2.5, 10.5281/zenodo.1173536)^88^.

### Western immunoblotting

All lysates were prepared at 4 °C and first processed using a Fast Prep-24 5G Benchtop Homogenizer (MP Biomedicals) using a 4:1 ratio of lysate to 0.5-mm zirconia/silicon beads (BioSpec). For histone methylation analysis, embryos were lysed in RIPA buffer. Lysates were treated with 5 µl of benzonase (Sigma) for 1 h at 4 °C with rotation. For acetylation analysis, embryos were lysed in TAP buffer (150 mM NaCl, 20 mM Tris-HCl, pH 7.5, 0.5% NP-40, 1 mM EDTA, 10% glycerol, 2× cOmplete-EDTA-free protease inhibitors (Roche), 1× Deacetylase Inhibitor (Active Motif) and 1 mM DTT) and sonicated using a Bioruptor Plus (Diagenode) for 14 cycles, 15 s on, 30 s off. TAP lysates were cleared at 21,000*g* for 10 min. Then, 10 µg of total protein was separated on Bis-Tris gels (Bio-Rad or Invitrogen) using MES buffer and transferred to 0.2-µm PVDF (Bio-Rad). Membranes were blocked with Protein-Free Blocking Buffer (Pierce) or PBS plus 0.5% Tween-20 (PBS-T) with 5% powdered milk (Sigma). Antibody dilutions: 1:2,000 mouse anti-H3K9me2 (MBL), 1:1,000 rabbit anti-RFP antibody, preadsorbed (Rockland), 1:100,000 mouse anti-H3K9me3 (MBL), 1:60,000 rabbit anti-H3K9me1 (ab8896; Abcam), 1:40,000 mouse anti-H3K9acetyl (a gift from H. Kimura), 1:40,000 rabbit anti-acetyl-Histone H3 (Millipore), 1:2,000 rabbit anti-acetyl-Histone H4 (Millipore), 1:20,000 rabbit anti-H2B (Abcam) and 1:40,000 rabbit anti-MRG-1 (49130002; Novus Biologicals). After overnight rotation with antibodies diluted in blocking buffer at 4 °C, blots were washed three times in PBS-T, re-blocked and exposed to HRP-conjugated secondary antibodies for 1 h (goat anti-mouse IgG HRP (1:10,000; Jackson ImmunoResearch 115-035-146), goat rabbit IgG HRP (1:20,000; Jackson ImmunoResearch 111-035-144)). After three further washes in PBS-T, ECL (Millipore) signal was detected with an Imager 600 (GE).

### Mass spectrometry

For immunoprecipitation experiments, embryos isolated by bleaching gravid adults were lysed at 4 °C in TAP buffer. Proteins from homogenized, sonicated and cleared TAP lysates (above) were immunoprecipitated with anti-FLAG-M2 beads (Sigma). Proteins were digested, bound to beads and subjected to LC–MS/MS exactly as described previously^14^. Intensity-based absolute quantification values were used as a measure of protein abundance in immunoprecipitation^89^. For H3K9 post-translational modification profiling, we employed the Histone Purification Kit Mini (Active Motif). Embryos isolated by bleaching were homogenized as above in histone extraction buffer. After overnight extraction at 4 °C with rotation, we proceeded with histone purification according to the manufacturer’s instructions. Purified histones were propionylated twice before and once after overnight trypsin digestion to prevent cleavage at lysine residues and facilitate separation of peptides. Samples were dried in a speedvac and resuspended in 0.1% trifluoroacetic acid (TFA), 2% acetonitrile. LC–MS was performed as follows: samples were loaded onto a PepMap 100 C18 2-cm trap (Thermo Fisher) using an EASY nLC-1000 system (Thermo Fisher). On-line peptide separation was then performed on a 15-cm EASY-Spray C18 column (ES801, Thermo Fisher) by applying a linear gradient of increasing acetonitrile concentration at a flow rate of 150 nl min^−1^. An Orbitrap Fusion Lumos Tribrid mass spectrometer (Thermo Fisher Scientific) was operated by alternating between MS1 survey scan mode, recording spectra at 120,000 resolution in the Orbitrap, and data-independent parallel reaction monitoring (PRM) mode, by targeting previously identified doubly charged histone H3 peptide isoforms of the sequence ‘KSTGGKAPR’ (9–17) containing lysines K9 and K14 (H3K9_K14me0 at *m*/*z* = 507.2873, H3K9me2K14ac at *m*/*z* = 514.2951, H3K9acK14ac at *m*/*z* = 521.2847, H3K9acK14me0 at *m*/*z* = 28.2926, H3K9me3K14me0 at *m*/*z* = 528.3107, H3K9me1K14ac at *m*/*z* = 535.3004, H3K9me1K14me0 at *m*/*z* = 542.3082, H3K4me0 at *m*/*z* = 522.2982, with ‘me0’ = in vitro propionylated, me1 = methyl + in vitro propionylated), and two endogenously unmodified reference peptide precursors (YRPGTVALR (41–49) at *m*/*z* = 544.8109 and YQKSTELLIR_K56me0 (54–63, with K56me0 = in vitro propionylated lysine) at *m*/*z* = 681.8817) for higher-energy collisional dissociation (HCD) fragmentation at a normalized collision energy of 33%, recorded at 15,000 resolution in the Orbitrap analyzer. Acquired data from strains indicated in Supplementary Table 1 were loaded into Skyline software (v.20.2.0.343)^90^, diagnostic fragment (*z* = 1) ions y6, y7 and y8 for each histone H3K9 peptide isoform (mark) of interest were selected and retention time windows were manually chosen to avoid quantifying background noise from coeluting isoforms or other unrelated analytes. Integrated fragment ion abundances were aggregated by peptide and histone mark for all samples and replicates and then divided by the total sum of all histone H3 peptide abundances per sample.

### Developmental phenotypes

For brood size, L4 animals grown from egg at the indicated temperature were singled out onto individual plates. They were transferred to fresh plates every 24 h until egg laying ceased, after which their total progeny was counted. For developmental rate, synchronized L1s were plated and the numbers of L4 and pre-L4 (delayed) animals were counted 42 h post feeding.

### RNA-seq

RNA was extracted from early embryos using Trizol as previously described^6^. Embryos were freeze-cracked five times, then RNA was extracted with choloroform followed by isopropanol precipitation. Further purification was performed with the RNA Clean and Concentrator kit (Zymo). Libraries were produced using the Smart-Seq2 mRNA sequencing kit (Illumina). Ribosomal RNA was depleted using Ribo-Zero Gold kit (Epicentre), and libraries were produced using the Total RNA Sequencing Scriptseq kit (Illumina). Equimolar amounts of indexed libraries were pooled and sequenced on a HiSeq 2500 (Illumina).

Reads were analyzed as described previously^6^. Adapters were trimmed using Trimmomatic v.0.39. Reads were aligned to the *C. elegans* genome (ce10) with the R package QuasR v.1.22.0 (www.bioconductor.org/packages/2.12/bioc/html/QuasR.html; ref. ^91^). The command ‘proj < -qAlign(‘samples.txt’,‘BSgenome. Celegans.UCSC.ce10’, splicedAlignment = TRUE)’ instructs hisat2 (ref. ^92^) to align using default parameters, considering unique reads for genes and genome-wide distribution. Count tables of reads mapping within annotated exons in WormBase (WS220) were constructed using the qCount function of the QuasR package to quantify the number of reads in each window (qCount(proj,GRange_object,orientation = ‘same’)) and normalized by division by the total number of reads in each library and multiplied by the average library size. Transformation into log_2_ space was performed after the addition of a pseudocount of 8 to minimize large changes in abundance FC caused by low count numbers. The EdgeR package v.3.24 was applied to select genes with differential transcript abundances between indicated genotypes (contrasts) based on FDRs for genes. Replica correlations are shown in Extended Data Fig. 6f,g. Annotation of tissue and cell type expression is based on annotation tables provided by the tissue atlas^93^.

### ChIP

ChIP experiments were performed as previously described^6^. In brief: early embryos were harvested from synchronized animals in three replicates. Then, 40 μg of chromatin was incubated overnight with 2 μg of anti-H3K9me2 (MBL), anti-H3K27ac (Abcam, ab177178), anti-H3K9me1 (Abcam, ab176880) or anti-acetyl-Histone H3 (Millipore) antibody coupled to Dynabeads Sheep Anti-Rabbit IgG (Invitrogen), in FA buffer (50 mM HEPES/KOH pH 7.5, 1 mM EDTA, 1% Triton X-100, 0.1% sodium deoxycholate, 150 mM NaCl)) containing 1% SDS. For MET-2::mCherry ChIP, 100 μg of chromatin was incubated with RFP-Trap magnetic agarose (Chromotek). Antibody-bound chromatin was washed 3 × 5 min with FA buffer; 5 min with FA buffer with 1 M NaCl; 10 min with FA buffer with 500 mM NaCl; 5 min with TEL buffer (0.25 M LiCl, 1% NP-40, 1% sodium deoxycholate, 1 mM EDTA, 10 mM Tris-HCl, pH 8.0) and 2 × 5 min with Tris-EDTA-buffer. Complexes were eluted in 1% SDS in Tris-EDTA buffer with 250 mM NaCl at 65 °C for 15 min. Samples and inputs were treated with 20 μg of RNAse A for 30 min at 37 °C and 20 μg of proteinase K for 1 h at 55 °C. Crosslinks were reversed by overnight incubation at 65 °C. DNA was subsequently purified using Zymo DNA purification columns (Zymo Research). Primers used in quantitative PCR are listed in Supplementary Table 2.

Libraries were prepared as previously described^6^ using the NEBNext Ultra DNA Library Prep kit for Illumina (NEB no. 7370) and the NEBNext Multiplex Oligos for Illumina (NEB no. E7335), according to the manufacturer’s recommendations, without size selection. Libraries were indexed and amplified using 12 PCR cycles, following the manufacturer’s recommendations. Libraries were further purified with Agencourt AmPure XP beads (Beckman no. A63881). Library size range and concentration were determined using a BioAnalyzer 2100 (Agilent Technologies) and Qubit (Invitrogen) instrument, respectively. Equimolar amounts of indexed libraries were pooled and sequenced on a HiSeq 2500 (Illumina) in rapid mode (Paired-End 50). Reads were aligned to the *C. elegans* genome (ce10) with the R package QuasR v.1.22.0 (www.bioconductor.org/packages/2.12/bioc/html/QuasR.html). The command ‘proj < -qAlign(‘samples.txt’,‘BSgenome. Celegans.UCSC.ce10’)’ instructs bowtie to align using the parameters ‘-m 1–best–strata–phred33-quals’, considering unique reads for genes and genome-wide distribution.

Read density was calculated by tiling the genome into 500-base-pair (bp) nonoverlapping windows and using the qCount function of the QuasR package to quantify the number of reads in each window (qCount(proj,GRange_object,orientation = ‘any’)). H3K9me-positive domains were determined as regions with a consecutive enrichment of H3K9me2 over input. For gene quantification, gene annotation from WormBase was used (WS220). Quantitation for each gene was performed by counting the reads overlapping the exons. Genome annotation was based on BSgenome.Celegans.UCSC.ce10 package (https://bioconductor.org/packages/release/data/annotation/html/BSgenome.Celegans.UCSC.ce10.html). Differences in read depths between samples were normalized by dividing each sample by total reads and multiplying by average library size. The various count tables used throughout this study were normalized according to the total genome count. log_2_ expression levels were determined after addition of a pseudocount of 2 (*y* = log_2_(*x* + 2)) to minimize large changes in FC caused by low count numbers. Results are displayed as the mean enrichment of immunoprecipitation − input (log_2_). H3K27ac peaks were called using MACS2 (ref. ^94^) with the following parameters: -g 93260000 --broad. Differential peaks among the genotypes were called using the Diffbind package v.3.0.11 (http://bioconductor.org/packages/release/bioc/html/DiffBind.html)^95^, normalizing on the total read counts and using the implemented DESeq2 analysis with standard parameters. Read density was calculated by tiling the genome into 500-bp nonoverlapping windows, or specifically at promoters (defined as 1 kilobase upstream and 100 bp downstream of transcription start site), and using the qCount function of the QuasR package to quantify the number of reads in each window (qCount(proj,GRange_object,orientation = ‘any’)). Differences in read depths between samples were normalized by dividing each sample by total reads and multiplying by average library size. The various count tables used throughout this study were normalized according to the total genome count.

### Reporting Summary

Further information on research design is available in the Nature Research Reporting Summary linked to this article.

## Online content

Any methods, additional references, Nature Research reporting summaries, source data, extended data, supplementary information, acknowledgements, peer review information; details of author contributions and competing interests; and statements of data and code availability are available at 10.1038/s41594-021-00712-4.

## Supplementary information

Supplementary Information Supplementary Tables 1 and 2.

Reporting Summary.

## Data Availability

All genome-wide datasets from this study have been uploaded to the Gene Expression Omnibus (GEO) under accession number GSE168925, GSE122341, and to the Sequence Read Archive (SRA) under SRP080806. Other numerical measurements are provided as Source data with this paper.

## Source data

Source Data Fig. 1 Statistical source data.
Source Data Fig. 1 Unprocessed western blots.
Source Data Fig. 2 Statistical source data.
Source Data Fig. 2 Unprocessed western blots.
Source Data Fig. 3 Statistical source data.
Source Data Fig. 3 Unprocessed western blots.
Source Data Fig. 4 Statistical source data.
Source Data Fig. 5 Statistical source data.
Source Data Fig. 5 Unprocessed western blots.
Source Data Extended Data Fig. 1 Statistical source data.
Source Data Extended Data Fig. 1 Unprocessed western blots.
Source Data Extended Data Fig. 2 Statistical source data.
Source Data Extended Data Fig. 2 Unprocessed western blots.
Source Data Extended Data Fig. 3 Statistical source data.
Source Data Extended Data Fig. 3 Unprocessed western blots.
Source Data Extended Data Fig. 4 Statistical source data.
Source Data Extended Data Fig. 5 Statistical source data.
Source Data Extended Data Fig. 6 Statistical source data.
Source Data Extended Data Fig. 6 Unprocessed western blots.
