# Author Correction: SETDB1-like MET-2 promotes transcriptional silencing and development independently of its H3K9me-associated catalytic activity

**DOI:** 10.1038/s41594-022-00776-w

**Published:** 2022-04-15

**Authors:** Colin E. Delaney, Stephen P. Methot, Veronique Kalck, Jan Seebacher, Daniel Hess, Susan M. Gasser, Jan Padeken

**Affiliations:** 1 Friedrich Miescher Institute for Biomedical Research, Basel, Switzerland; 2 Faculty of Sciences, University of Basel, Basel, Switzerland; 3 Present Address: Fondation ISREC, Lausanne, Switzerland

**Keywords:** Epigenomics, Gene silencing

Correction to: *Nature Structural & Molecular Biology* 10.1038/s41594-021-00712-4, published online 31 January 2022.

In the version of this article initially published, there was a typographical error in the abstract. The cofactor LIN-65 originally read “LIN-61.” The corrected sentence now reads “Our study suggests that the noncatalytic, focus-forming function of this SETDB1-like protein and its intrinsically disordered cofactor LIN-65 is physiologically relevant.” The change has been made to the HTML and PDF versions the article.

