# Supplementary material for: SETDB1-like MET-2 promotes transcriptional silencing and development independently of its H3K9me-associated catalytic activity
Source: Nat Struct Mol Biol. 2022 Jan 31;29(2):85–96. doi: 10.1038/s41594-021-00712-4 (PMC8850192; doi:10.1038/s41594-021-00712-4)
Supplement: Supplementary file 1 — Supplementary Tables 1 and 2. [file 41594_2021_712_MOESM1_ESM.pdf]

nature portfolio

https://doi.org/10.1038/s41594-021-00712-4

Supplementary information

SETDB1-like MET-2 promotes transcriptional silencing and development independently of its H3K9me-associated catalytic activity

In the format provided by the authors and unedited

Supplementary Table 1. *C elegans* strains used in this study

Strain	Genotype
GW1	N2 (WT)
GW638	*met-2(n4256) set-25(n5021)*
GW641	*set-25(n5021)*
GW799	*lin-61(tm2649)*
GW802	*lin-61(tm2649);met-2(n4256)*
GW907	*met-2(n4256)*
GW1270	*nrde-3(tm1116)*
GW1419	*met-2::1xflag::mCherry(gw1419)*
GW1444	*set-25(n5021);nrde-3(tm1116)*
GW1465	*lin-65(gw1465)*
GW1573	*lin-65(gw1465);met-2::1xflag::mCherry(gw1419)*
GW1583	*met-2(n4256);nrde-3(tm1116)*
GW1618	*lin-65::1xflag::GFP(gw1578)*
GW1630	*arle-14(gw1584)*
GW1639	*arle-14(tm6748)*
GW1679	*met-2-CD::1xflag::mCherry(gw1660 gw1419)*
GW1680	*lin-65(gw1465);arle-14(gw1584)*
GW1684	*met-2(gw1660)*
GW1693	*met-2(gw1660) set-25(n5021)*
OG497	*lin-65(gw1465);arle-14(gw1584)*
GW1729	*lin-65::1xflag::GFP(gw1578);met-2(gw1660)*
GW1761	*drSi13 [hsf-1p::hsf-1::GFP::unc-54 3'UTR + Cbr-unc-119(+)] II;met-2(n4256)*
GW1791	*NLS::met-2::1xflag::mCherry(gw1786 gw1419)*
GW1792	*lin-65(gw1465);NLS::met-2::1xflag::mCherry(gw1786 gw1419)*
GW1812	WT
GW1813	*lin-65(gw1465)*
GW1814	*arle-14(tm6845)*
GW1815	*lin-65(gw1465);arle-14(tm6845)*
GW1839	*lin-65(gw1465);met-2(gw1660)*
GW1851	*arle-14(tm6845) set-25(n5021)*
GW1852	*arle-14(tm6845) met-2(gw1660)*
GW1873	*arle-14(tm6845) met-2-CD::1xflag::mCherry(gw1660 gw1419)*

Supplementary Table 2. Oligonucleotides used in this study

CRISPR oligos	
arle-14(gw1584) crRNA	ATTTGTGCGAGAACGTCAT
arle-14(gw1584) HDR oligo	cctactaaatttctgaaccaataattctaaaaatgagctTCTAATGATGTCAACAGATCATCAAGATTAGAAAATGGTTGTAGCATTATCAAAGGTTcgttctcgcacaaattgccggtctggcgaaagc
	uppercase = modified sequence, underline = start codon, red = nonsense mutations
met-2(gw1660) crRNA	CGTGTTGAACGTGCACATT
met-2(gw1660) HDR oligo	gatacaatcataaattttcgataactttcagattcttgaatcactct**GCA**GATCCGAACGTTCATGTGCAGCATGtcatgtacgatacgcatgatcttcgtcttccatgg
	uppercase = modified sequence, bold = C1237A mutation
met-2(gw1786) crRNA	GTTATTCGATGGTTCTTGT
met-2(gw1786) HDR oligo	gcattgcctcaattaatttttatctaatattcagatg**CCAGCCGCCAAGCGTGTCAAGCTCGAC**GATcaacaagaaccatcgaataacgtagatacgagcag
	uppercase = modified sequence, bold = NLS tag (Mutlu et al, 2018), underline = start codon
ChIP primers	
col-166 forward	TCTGACGAGAAACAACTCGAAAT
col-166 reverse	GAGACGGCAACTCCAACAAGA
C55C3.3 forward	GAGCGATCCCGAAGTGTGAT
C55C3.3 reverse	TTGGATTGGAGCACGATTGC
F10D11.6 forward	TGGAGCTCTTCCACAAACTGT
F10D11.6 reverse	TGAAAATGAACATACAGTTCCACCT
bcl-11 forward	TGTGGTCGTTCAACATCAGC
bcl-11 reverse	AACATTTGCGGGCTCTTTGC
glf-1 forward	CAACACACAAATCACTGCTCCA
glf-1 reverse	TGATCAACCCAACTGCTAATTCTG
grl-16 forward	CACATTCACCCTCACCCACA
grl-16 reverse	TGTGGCTCGACAACAGTCTC
nep-17 forward	TAGCACGGACATTCACTGGC
nep-17 reverse	GGTCAGGCTAGGGCTTGAAA
unc-119 forward	CCACACCACCTCTAATCTCC
unc-119 reverse	TCATTTCTCTGCGTCTTCCT
