# Supplementary material for: SETDB1-like MET-2 promotes transcriptional silencing and development independently of its H3K9me-associated catalytic activity
Source: Nat Struct Mol Biol. 2022 Jan 31;29(2):85–96. doi: 10.1038/s41594-021-00712-4 (PMC8850192; doi:10.1038/s41594-021-00712-4)
Supplement: Supplementary file 2 — Reporting Summary. [file 41594_2021_712_MOESM2_ESM.pdf]

nature portfolio

Corresponding author(s): Susan Gasser

Last updated by author(s): Jun 29, 2021

Reporting Summary

Nature Portfolio wishes to improve the reproducibility of the work that we publish. This form provides structure for consistency and transparency in reporting. For further information on Nature Portfolio policies, see our Editorial Policies and the Editorial Policy Checklist.

Statistics

For all statistical analyses, confirm that the following items are present in the figure legend, table legend, main text, or Methods section.

n/a	Confirmed	
☐	☒	The exact sample size (*n*) for each experimental group/condition, given as a discrete number and unit of measurement
☐	☒	A statement on whether measurements were taken from distinct samples or whether the same sample was measured repeatedly
☐	☒	The statistical test(s) used AND whether they are one- or two-sided<br>*Only common tests should be described solely by name; describe more complex techniques in the Methods section.*
☐	☒	A description of all covariates tested
☐	☒	A description of any assumptions or corrections, such as tests of normality and adjustment for multiple comparisons
☐	☒	A full description of the statistical parameters including central tendency (e.g. means) or other basic estimates (e.g. regression coefficient) AND variation (e.g. standard deviation) or associated estimates of uncertainty (e.g. confidence intervals)
☐	☒	For null hypothesis testing, the test statistic (e.g. *F*, *t*, *r*) with confidence intervals, effect sizes, degrees of freedom and *P* value noted<br>*Give P values as exact values whenever suitable.*
☒	☐	For Bayesian analysis, information on the choice of priors and Markov chain Monte Carlo settings
☒	☐	For hierarchical and complex designs, identification of the appropriate level for tests and full reporting of outcomes
☐	☒	Estimates of effect sizes (e.g. Cohen's *d*, Pearson's *r*), indicating how they were calculated

Our web collection on statistics for biologists contains articles on many of the points above.

Software and code

Policy information about availability of computer code

Data collection	For data collection and conversion to fastq format RTA 1.18.64 (HiSeq2500), RTA 2.4.11 (NextSeq500) and bcl2fastq2 v2.17 were used. Microscopic mages were acquired on spinning disk multipoint confocal microscopes using Visiview software (Visitron): (1) AxioImager M1 with Yokogawa CSU-X1 scan head, A plan-NEOFLUAR 100×/1.45 oil, Rolera Thunder Back Illuminated EM-CCD (Q Imaging) and VisiView v.4.4.0.14. (2) Nikon Ti2-E Eclipse with Yokagawa CSU W1 scan head, CFI P-Apo Lambda 60x/1.4 oil, iXon-Ultra-888 Back illuminated EM-CCD (Andor) and Visiview v4.5.0.10. GFP/NeonGreen and RFP/mCherry/Rhodamine flurophores were excited using a Toptica iBeam Smart 488-nm and 561-nm lasers, respectively. Western images were acquired with an Amersham Imager 600. Mass spectrometry data was acquired with an EASY nLC-1000 system (Thermo Fisher).
Data analysis	R package QuasR v1.22.0, EdgeR package v3.24, BSgenome.Celegans.UCSC.ce10 v1.0 package, Trimmomatic v0.39, bowtie2 v2.3.5.1, Bismark program v0.22.3 (https://github.com/FelixKrueger/Bismark), Diffbind package v 3.0.11, MACS2 version 2.2.7.1, plots were created using ggplot2 v3.3.5,Microscopy images were analysed using the Fiji/ImageJ v1.53c and the KNIME Analytics Platform v4.3.3 software, with TrackMate v0.2.5., histone PTMs were analyzed with Skyline software (version 20.2.0.343).

For manuscripts utilizing custom algorithms or software that are central to the research but not yet described in published literature, software must be made available to editors and reviewers. We strongly encourage code deposition in a community repository (e.g. GitHub). See the Nature Portfolio guidelines for submitting code & software for further information.

Data

Policy information about availability of data

All manuscripts must include a data availability statement. This statement should provide the following information, where applicable:

- Accession codes, unique identifiers, or web links for publicly available datasets
- A description of any restrictions on data availability
- For clinical datasets or third party data, please ensure that the statement adheres to our policy

All genome wide datasets (RNA-seq, ChIP-seq) performed for this study have been uploaded to the Gene Expression Omnibus (GEO) under accession number GSE168925. Previous published and reanalyzed data is accessible as GSE122341, and the Sequence Read Archive (SRA) SRP080806.

Field-specific reporting

Please select the one below that is the best fit for your research. If you are not sure, read the appropriate sections before making your selection.

☒ Life sciences ☐ Behavioural & social sciences ☐ Ecological, evolutionary & environmental sciences

For a reference copy of the document with all sections, see nature.com/documents/nr-reporting-summary-flat.pdf

Life sciences study design

All studies must disclose on these points even when the disclosure is negative.

Sample size	Statistical power calculations were used to determine population sizes. All experiments were conducted with at least two independent biological<br>replicates. When the data showed a clear trend but was not yet conclusive, we performed additional replicates to examine the trend. For full list of strains used in this study see supplementary information.
Data exclusions	no data was excluded.
Replication	Experimental data was reliably reproduced. Each experiment was performed at least twice and up to four times.
Randomization	samples were grouped by genotype.
Blinding	Group allocation according to genotype was done before data collection. Measurements were automated whenever possible. Manually scored images were performed without knowledge of the genotype.

Reporting for specific materials, systems and methods

We require information from authors about some types of materials, experimental systems and methods used in many studies. Here, indicate whether each material, system or method listed is relevant to your study. If you are not sure if a list item applies to your research, read the appropriate section before selecting a response.

Materials & experimental systems

n/a	Involved in the study	
☐	☒	Antibodies
☒	☐	Eukaryotic cell lines
☒	☐	Palaeontology and archaeology
☐	☒	Animals and other organisms
☒	☐	Human research participants
☒	☐	Clinical data
☒	☐	Dual use research of concern

Methods

n/a	Involved in the study	
☐	☒	ChIP-seq
☒	☐	Flow cytometry
☒	☐	MRI-based neuroimaging

Antibodies

Antibodies used	mouse anti-H3K9me3 (MABI0318 (MBL; Kimura et al., 2008), mouse anti-H3K9me2 (MABI0317 (MBL; Kimura et al., 2008), rabbit anti-H3K9me1 (abcam ab176880), mouse anti-H3K27ac (H. Kimura, Kimura et al., 2008), mouse anti-H3K9ac (H. Kimura, Kimura et al., 2008), and recombinant anti-H3K27ac (ab177178), rabbit anti-MRG-1 (49130002, Novus Biologicals), rabbit anti-H4 (Abcam #ab10158), rabbit anti-RFP antibody, pre-adsorbed (Rockland 600-401-379), rabbit anti-acetyl-Histone H3 (Millipore 06-599), rabbit ant-acetyl-Histone H4 (06-866 Millipore), rabbit anti-H2B (abcam ab1790), goat anti-mouse IgG HRP (Jackson ImmunoResearch 115-035-146), goat rabbit IgG HRP (Jackson Immuno Research 111-035-144), donkey anti-rabbit Alexa Fluor 555 (Thermo Fischer, # A-31572)

Validation

For mouse anti-H3K9me3, H3K9me2, H3K9ac, H3K27ac, see: Kimura H, Hayashi-Takanaka Y, Goto Y, Takizawa N, Nozaki N. The organization of histone H3 modifications as revealed by a panel of specific monoclonal antibodies. Cell Struct Funct. 2008;33(1):61-73. doi:10.1247/csf.07035 and Zeller P, Padeken J, van Schendel R, Kalck V, Tijsterman M, Gasser SM. Histone H3K9 methylation is dispensable for Caenorhabditis elegans development but suppresses RNA:DNA hybrid-associated repeat instability. Nat Genet. 2016;48(11):1385-1395. doi:10.1038/ng.3672
H3K27ac (ab177178) was tested on a peptide array, ChIP enrichment at known targets, immunostaining and WB on TSA treated cells, for references see: https://www.abcam.com/histone-h3-acetyl-k27-antibody-ep16602-chip-grade-ab177178.html
The rabbit anti-MRG-1 (#49130002, Novus Biologicals) was validated by Novus Biologicals in WB and Immunofluorescence against wild-type and MRG-1 deficient cells. Source: (https://www.novusbio.com/products/mrg-1-antibody_49130002#ReviewsSection)
The rabbit anti-RFP antibody, pre-adsorbed (Rockland 600-401-379) was tested for specificity as follows: "This product was prepared from monospecific antiserum by immunoaffinity chromatography using Red Fluorescent Protein (Discosoma) coupled to agarose beads followed by solid phase adsorption(s) to remove any unwanted reactivities. Expect reactivity against RFP and its variants: mCherry, tdTomato, mBanana, mOrange, mPlum, mOrange and mStrawberry. Assay by immunoelectrophoresis resulted in a single precipitin arc against anti-Rabbit Serum and purified and partially purified Red Fluorescent Protein (Discosoma). No reaction was observed against Human, Mouse or Rat serum proteins." Source: https://rockland-inc.com/store/Antibodies-to-GFP-and-Antibodies-to-RFP-600-401-379-O4L_24299.aspx
The rabbit anti-H3K9me1 (abcam ab176880) was tested on a peptide array, ChIP enrichment at known targets, and immunostaining. Source:https://www.abcam.com/histone-h3-mono-methyl-k9-antibody-epr16989-chip-grade-ab176880.html
The rabbit anti-H4 (Abcam #ab10158) was tested by the manufacturer by western, ChIP and IHC. Source: https://www.abcam.com/histone-h4-antibody-chip-grade-ab10158.html
The rabbit anti-acetyl-Histone H3 (Millipore 06-599) has been shown to work in WB, ICC, ChIP, ChIP-seq. Source: https://www.merckmillipore.com/CH/en/product/Anti-acetyl-Histone-H3-Antibody,MM_NF-06-599
The rabbit anti-acetyl-Histone H4 (06-866) is validated in ChIP, WB, ICC to detect acetyl-Histone H4. Source: https://www.merckmillipore.com/CH/en/product/Anti-acetyl-Histone-H4-Antibody,MM_NF-06-866
The rabbit anti-H2B (abcam ab1790) was validated by the manufacturer by Western, ChIP, and immunohistochemistry. Source: https://www.abcam.com/histone-h2b-antibody-chip-grade-ab1790.html

Animals and other organisms

Policy information about studies involving animals; ARRIVE guidelines recommended for reporting animal research

Laboratory animals

The manuscript utilized Caenorhabditis elegans (variant Bristol) as non-vertebrate model organism. A list of specific strains is attached as Supplementary information. Animals used for this study were hermaphrodites. Ages/developmental stages are indicated in the manuscript (ranging from early embryos (<200 cells), to 1 day old adults)

Wild animals

study did not use wild animals

Field-collected samples

this study did not use field-collected samples

Ethics oversight

As a non-vertebrate, C. elegans does not fall under the Directive 2010/63/EU of the European Parliament and of the Council of 22 September 2010 on the protection of animals used for scientific purposes: http://eur-lex.europa.eu/LexUriServ/LexUriServ.do?uri=OJ:L:2010:276:0033:0079:en:PDF

Note that full information on the approval of the study protocol must also be provided in the manuscript.

ChIP-seq

Data deposition

☒ Confirm that both raw and final processed data have been deposited in a public database such as GEO.

☒ Confirm that you have deposited or provided access to graph files (e.g. BED files) for the called peaks.

Data access links
May remain private before publication.

To review GEO accession GSE168925:
Go to https://www.ncbi.nlm.nih.gov/geo/query/acc.cgi?acc=GSE168925
Enter token grsjgciexlcvfan into the box

Files in database submission

ChIP_H3K27ac_hs_2651F28-1_R1_001.fastq.gz
ChIP_H3K27ac_hs_2651F28-1_R2_001.fastq.gz
ChIP_H3K27ac_hs_2651F28-2_R1_001.fastq.gz
ChIP_H3K27ac_hs_2651F28-2_R2_001.fastq.gz
ChIP_H3K27ac_hs_2651F29-1_R1_001.fastq.gz
ChIP_H3K27ac_hs_2651F29-1_R2_001.fastq.gz
ChIP_H3K27ac_hs_2651F29-2_R1_001.fastq.gz
ChIP_H3K27ac_hs_2651F29-2_R2_001.fastq.gz
ChIP_H3K27ac_hs_2651F30-1_R1_001.fastq.gz
ChIP_H3K27ac_hs_2651F30-1_R2_001.fastq.gz
ChIP_H3K27ac_hs_2651F30-2_R1_001.fastq.gz
ChIP_H3K27ac_hs_2651F30-2_R2_001.fastq.gz
ChIP_H3K27ac_met2_2651F31-1_R1_001.fastq.gz
ChIP_H3K27ac_met2_2651F31-1_R2_001.fastq.gz

ChIP_H3K27ac_met2_2651F31-2_R1_001.fastq.gz
ChIP_H3K27ac_met2_2651F31-2_R2_001.fastq.gz
ChIP_H3K27ac_met2_2651F32-1_R1_001.fastq.gz
ChIP_H3K27ac_met2_2651F32-1_R2_001.fastq.gz
ChIP_H3K27ac_met2_2651F32-2_R1_001.fastq.gz
ChIP_H3K27ac_met2_2651F32-2_R2_001.fastq.gz
ChIP_H3K27ac_met2_2651F33-1_R1_001.fastq.gz
ChIP_H3K27ac_met2_2651F33-1_R2_001.fastq.gz
ChIP_H3K27ac_met2_2651F33-2_R1_001.fastq.gz
ChIP_H3K27ac_met2_2651F33-2_R2_001.fastq.gz
ChIP_H3K27ac_met2cd_2651F34-1_R1_001.fastq.gz
ChIP_H3K27ac_met2cd_2651F34-1_R2_001.fastq.gz
ChIP_H3K27ac_met2cd_2651F34-2_R1_001.fastq.gz
ChIP_H3K27ac_met2cd_2651F34-2_R2_001.fastq.gz
ChIP_H3K27ac_met2cd_2651F35-1_R1_001.fastq.gz
ChIP_H3K27ac_met2cd_2651F35-1_R2_001.fastq.gz
ChIP_H3K27ac_met2cd_2651F35-2_R1_001.fastq.gz
ChIP_H3K27ac_met2cd_2651F35-2_R2_001.fastq.gz
ChIP_H3K27ac_met2cd_2651F36-1_R1_001.fastq.gz
ChIP_H3K27ac_met2cd_2651F36-1_R2_001.fastq.gz
ChIP_H3K27ac_met2cd_2651F36-2_R1_001.fastq.gz
ChIP_H3K27ac_met2cd_2651F36-2_R2_001.fastq.gz
ChIP_H3K27ac_wt_2651F25-1_R1_001.fastq.gz
ChIP_H3K27ac_wt_2651F25-1_R2_001.fastq.gz
ChIP_H3K27ac_wt_2651F25-2_R1_001.fastq.gz
ChIP_H3K27ac_wt_2651F25-2_R2_001.fastq.gz
ChIP_H3K27ac_wt_2651F26-1_R1_001.fastq.gz
ChIP_H3K27ac_wt_2651F26-1_R2_001.fastq.gz
ChIP_H3K27ac_wt_2651F26-2_R1_001.fastq.gz
ChIP_H3K27ac_wt_2651F26-2_R2_001.fastq.gz
ChIP_H3K27ac_wt_2651F27-1_R1_001.fastq.gz
ChIP_H3K27ac_wt_2651F27-1_R2_001.fastq.gz
ChIP_H3K27ac_wt_2651F27-2_R1_001.fastq.gz
ChIP_H3K27ac_wt_2651F27-2_R2_001.fastq.gz
ChIP_H3K9me2_wt_2081F5-1_R1_001.fastq.gz
ChIP_H3K9me2_wt_2081F5-1_R2_001.fastq.gz
ChIP_H3K9me2_wt_2081F5-2_R1_001.fastq.gz
ChIP_H3K9me2_wt_2081F5-2_R2_001.fastq.gz
ChIP_H3K9me2_wt_2081F6-1_R1_001.fastq.gz
ChIP_H3K9me2_wt_2081F6-1_R2_001.fastq.gz
ChIP_H3K9me2_wt_2081F6-2_R1_001.fastq.gz
ChIP_H3K9me2_wt_2081F6-2_R2_001.fastq.gz
ChIP_H3K9me2_wt-hs_2081F7-1_R1_001.fastq.gz
ChIP_H3K9me2_wt-hs_2081F7-1_R2_001.fastq.gz
ChIP_H3K9me2_wt-hs_2081F7-2_R1_001.fastq.gz
ChIP_H3K9me2_wt-hs_2081F7-2_R2_001.fastq.gz
ChIP_H3K9me2_wt-hs_2081F8-1_R1_001.fastq.gz
ChIP_H3K9me2_wt-hs_2081F8-1_R2_001.fastq.gz
ChIP_H3K9me2_wt-hs_2081F8-2_R1_001.fastq.gz
ChIP_H3K9me2_wt-hs_2081F8-2_R2_001.fastq.gz
H3K27ac_emb_500bp_unique.tab
H3K9me2_heatshock_500bp_unique.tab
input_H3K27ac_hs_2651F4-1_R1_001.fastq.gz
input_H3K27ac_hs_2651F4-1_R2_001.fastq.gz
input_H3K27ac_hs_2651F4-2_R1_001.fastq.gz
input_H3K27ac_hs_2651F4-2_R2_001.fastq.gz
input_H3K27ac_hs_2651F5-1_R1_001.fastq.gz
input_H3K27ac_hs_2651F5-1_R2_001.fastq.gz
input_H3K27ac_hs_2651F5-2_R1_001.fastq.gz
input_H3K27ac_hs_2651F5-2_R2_001.fastq.gz
input_H3K27ac_hs_2651F6-1_R1_001.fastq.gz
input_H3K27ac_hs_2651F6-1_R2_001.fastq.gz
input_H3K27ac_hs_2651F6-2_R1_001.fastq.gz
input_H3K27ac_hs_2651F6-2_R2_001.fastq.gz
input_H3K27ac_met2_2651F7-1_R1_001.fastq.gz
input_H3K27ac_met2_2651F7-1_R2_001.fastq.gz
input_H3K27ac_met2_2651F7-2_R1_001.fastq.gz
input_H3K27ac_met2_2651F7-2_R2_001.fastq.gz
input_H3K27ac_met2_2651F8-1_R1_001.fastq.gz
input_H3K27ac_met2_2651F8-1_R2_001.fastq.gz
input_H3K27ac_met2_2651F8-2_R1_001.fastq.gz
input_H3K27ac_met2_2651F8-2_R2_001.fastq.gz
input_H3K27ac_met2_2651F9-1_R1_001.fastq.gz
input_H3K27ac_met2_2651F9-1_R2_001.fastq.gz
input_H3K27ac_met2_2651F9-2_R1_001.fastq.gz
input_H3K27ac_met2_2651F9-2_R2_001.fastq.gz

input_H3K27ac_met2cd_2651F10-1_R1_001.fastq.gz
input_H3K27ac_met2cd_2651F10-1_R2_001.fastq.gz
input_H3K27ac_met2cd_2651F10-2_R1_001.fastq.gz
input_H3K27ac_met2cd_2651F10-2_R2_001.fastq.gz
input_H3K27ac_met2cd_2651F11-1_R1_001.fastq.gz
input_H3K27ac_met2cd_2651F11-1_R2_001.fastq.gz
input_H3K27ac_met2cd_2651F11-2_R1_001.fastq.gz
input_H3K27ac_met2cd_2651F11-2_R2_001.fastq.gz
input_H3K27ac_met2cd_2651F12-1_R1_001.fastq.gz
input_H3K27ac_met2cd_2651F12-1_R2_001.fastq.gz
input_H3K27ac_met2cd_2651F12-2_R1_001.fastq.gz
input_H3K27ac_met2cd_2651F12-2_R2_001.fastq.gz
input_H3K27ac_wt_2651F1-1_R1_001.fastq.gz
input_H3K27ac_wt_2651F1-1_R2_001.fastq.gz
input_H3K27ac_wt_2651F1-2_R1_001.fastq.gz
input_H3K27ac_wt_2651F1-2_R2_001.fastq.gz
input_H3K27ac_wt_2651F2-1_R1_001.fastq.gz
input_H3K27ac_wt_2651F2-1_R2_001.fastq.gz
input_H3K27ac_wt_2651F2-2_R1_001.fastq.gz
input_H3K27ac_wt_2651F2-2_R2_001.fastq.gz
input_H3K27ac_wt_2651F3-1_R1_001.fastq.gz
input_H3K27ac_wt_2651F3-1_R2_001.fastq.gz
input_H3K27ac_wt_2651F3-2_R1_001.fastq.gz
input_H3K27ac_wt_2651F3-2_R2_001.fastq.gz
input_H3K9me2_wt_2081F1-1_R1_001.fastq.gz
input_H3K9me2_wt_2081F1-1_R2_001.fastq.gz
input_H3K9me2_wt_2081F1-2_R1_001.fastq.gz
input_H3K9me2_wt_2081F1-2_R2_001.fastq.gz
input_H3K9me2_wt_2081F2-1_R1_001.fastq.gz
input_H3K9me2_wt_2081F2-1_R2_001.fastq.gz
input_H3K9me2_wt_2081F2-2_R1_001.fastq.gz
input_H3K9me2_wt_2081F2-2_R2_001.fastq.gz
input_H3K9me2_wt-hs_2081F3-1_R1_001.fastq.gz
input_H3K9me2_wt-hs_2081F3-1_R2_001.fastq.gz
input_H3K9me2_wt-hs_2081F3-2_R1_001.fastq.gz
input_H3K9me2_wt-hs_2081F3-2_R2_001.fastq.gz
input_H3K9me2_wt-hs_2081F4-1_R1_001.fastq.gz
input_H3K9me2_wt-hs_2081F4-1_R2_001.fastq.gz
input_H3K9me2_wt-hs_2081F4-2_R1_001.fastq.gz
input_H3K9me2_wt-hs_2081F4-2_R2_001.fastq.gz

Genome browser session
(e.g. UCSC)

no longer applicable

Methodology

Replicates

H3K9me2 ChIP-seq has 2 and H3K27ac ChIP-seq has 3 biological replica

Sequencing depth

All samples were sequenced paired end, reads are 50nt long.
sample sample total_reads unique_mapped_reads
input_wt_H3K27ac_1 19463818 16899918
input_wt_H3K27ac_2 24031198 20680726
input_wt_H3K27ac_3 27328862 23411210
input_wt_37°C_H3K27ac_1 29756504 26211456
input_wt_37°C_H3K27ac_2 24887740 22093746
input_wt_37°C_H3K27ac_3 32202920 28329818
input_met2_H3K27ac_1 33026772 29466448
input_met2_H3K27ac_2 73452934 65133682
input_met2_H3K27ac_3 24387396 21180014
input_met2cd_H3K27ac_1 28611264 25127156
input_met2cd_H3K27ac_2 29945856 25569318
input_met2cd_H3K27ac_3 29015292 25447510
ChIP_H3K27ac_wt_1 23744090 21361728
ChIP_H3K27ac_wt_2 21702430 19327992
ChIP_H3K27ac_wt_3 29361010 26141674
ChIP_H3K27ac_wt_37°C_1 31930890 28896446
ChIP_H3K27ac_wt_37°C_2 29505794 26876674
ChIP_H3K27ac_wt_37°C_3 30294944 27328522
ChIP_H3K27ac_met2_1 40299360 36470940
ChIP_H3K27ac_met2_2 34273814 31083804
ChIP_H3K27ac_met2_3 34366132 30633968
ChIP_H3K27ac_met2cd_1 33387986 29983456
ChIP_H3K27ac_met2cd_2 23864600 21121024
ChIP_H3K27ac_met2cd_3 30071748 27474318
input_wt_H3K9me2_20°C_1 22304730 9928436

	input_wt_H3K9me2_20°C_2 102361372 89301222<br>input_wt_H3K9me2_37°C_1 38797510 20253164<br>input_wt_H3K9me2_37°C_2 42039964 17224180<br>ChIP_wt_H3K9me2_20°C_1 39615190 25239790<br>ChIP_wt_H3K9me2_20°C_2 52035804 36370888<br>ChIP_wt_H3K9me2_37°C_1 70468564 56442676<br>ChIP_wt_H3K9me2_37°C_2 62287706 49209124
Antibodies	mouse anti H3K9me2 (MABI0317 (MBL; Kimura et al., 2008), recombinant anti-H3K27ac (ab177178)
Peak calling parameters	MACS2 version 2.2.7.1 was used for peak-calling with the parameters " -f BAM -g 93260000 --broad". Peaks were called for each IP sample vs. its matched input control sample.
Data quality	A q-value cutoff of 0.05 was applied to all Adnp ChIP replicates separately. Plotting -log10 q-values for each replicate against each other showed a good correlation, indicating reproducibility.
Software	R package Bioconductor is version 3.12, QuasR v1.30.0, Diffbind package v 3.0.11, Trimmomatic v0.39, bowtie2, Bismark program (https://github.com/FelixKrueger/Bismark)
