# Supplementary figures and images for: SETDB1-like MET-2 promotes transcriptional silencing and development independently of its H3K9me-associated catalytic activity
Source: Nat Struct Mol Biol. 2022 Jan 31;29(2):85–96. doi: 10.1038/s41594-021-00712-4 (PMC8850192; doi:10.1038/s41594-021-00712-4)

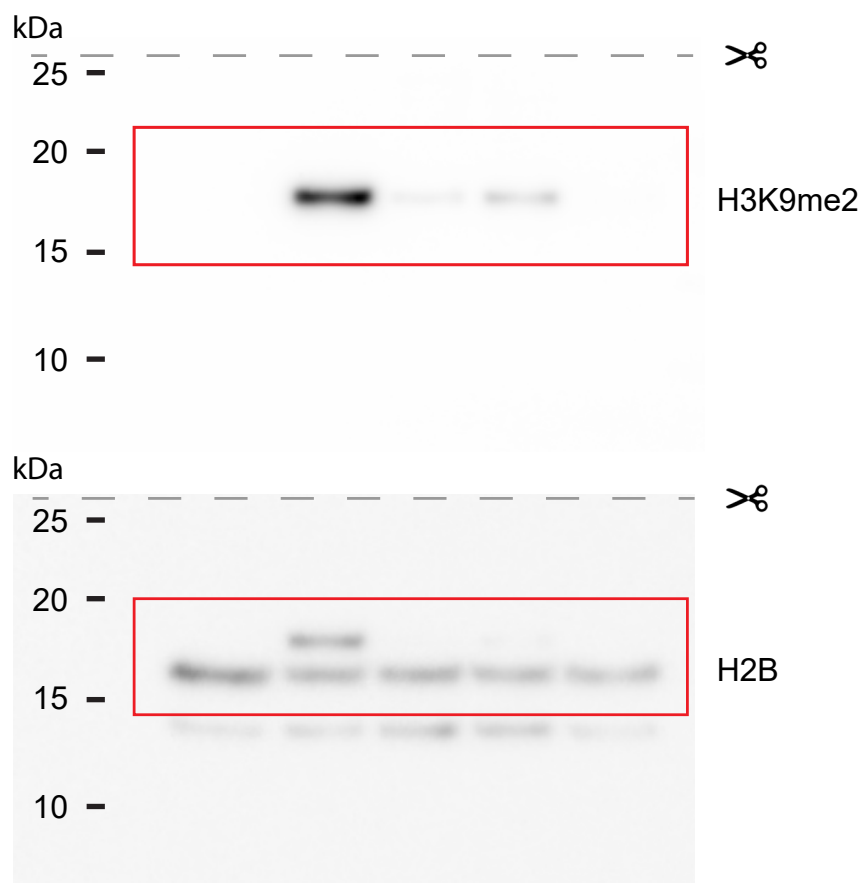

**Fig. 1f**

✂ = membrane cut prior to antibody incubation

Supplement: Source Data Fig. 1 — Unprocessed western blots. [file 41594_2021_712_MOESM4_ESM.pdf]

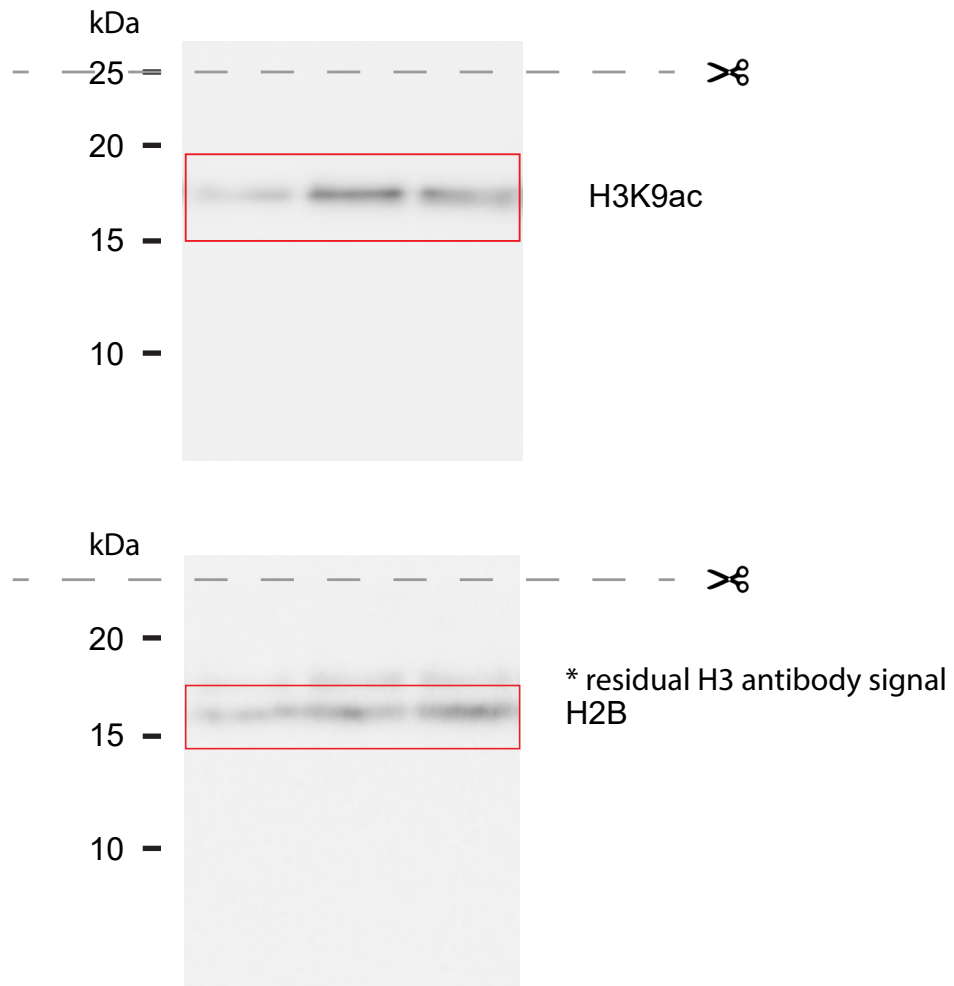

**Fig. 3c**

✂ = membrane cut prior to antibody incubation

Supplement: Source Data Fig. 3 — Unprocessed western blots. [file 41594_2021_712_MOESM8_ESM.pdf]

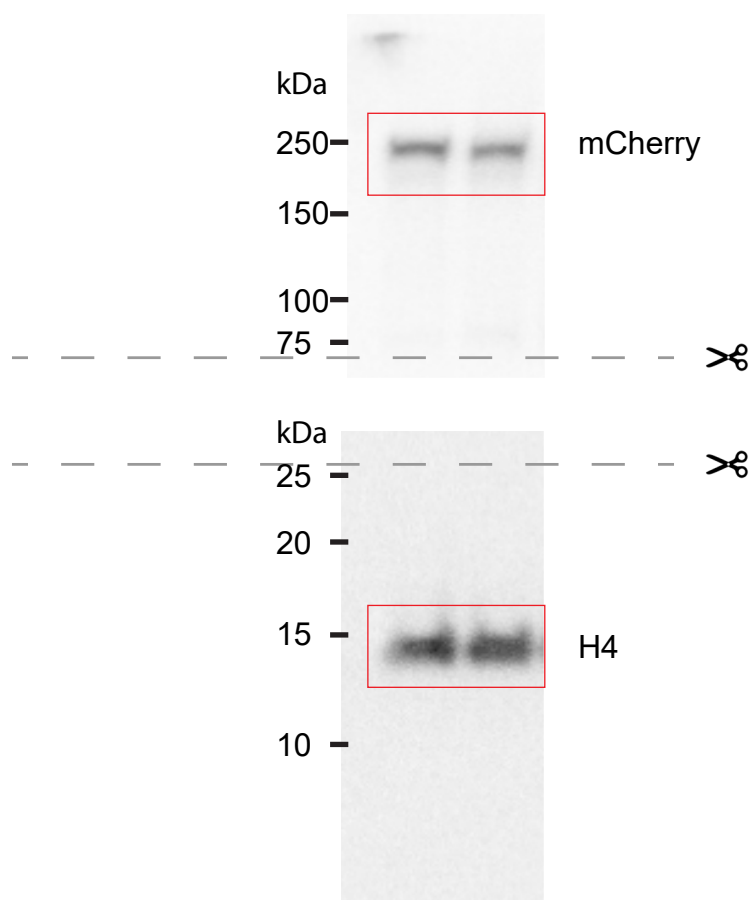

**Extended Data Figure 6c**

✂ = membrane cut prior to antibody incubation

Supplement: Source Data Extended Data Fig. 6 — Unprocessed western blots. [file 41594_2021_712_MOESM21_ESM.pdf]
