# Supplementary material for: SETDB1-like MET-2 promotes transcriptional silencing and development independently of its H3K9me-associated catalytic activity
Source: Nat Struct Mol Biol. 2022 Jan 31;29(2):85–96. doi: 10.1038/s41594-021-00712-4 (PMC8850192; doi:10.1038/s41594-021-00712-4)
Supplement: Source Data Fig. 2 — Unprocessed western blots. [file 41594_2021_712_MOESM6_ESM.pdf]

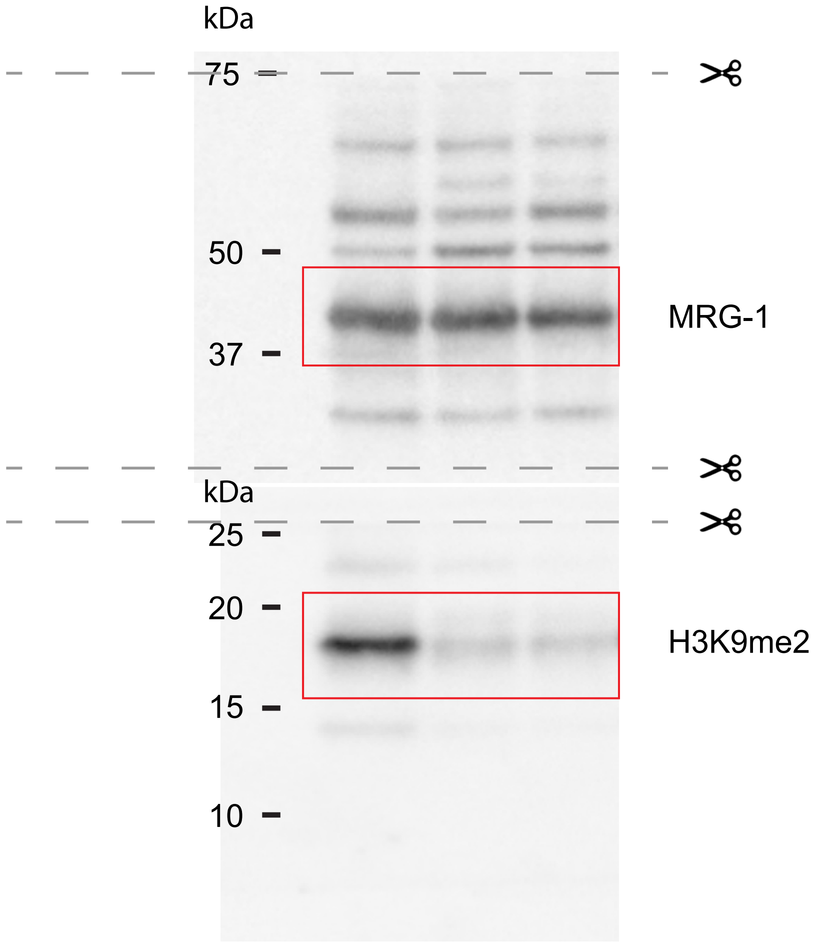

Fig. 2b

✂ = membrane cut prior to antibody incubation

Delaney et al
unprocessed blots

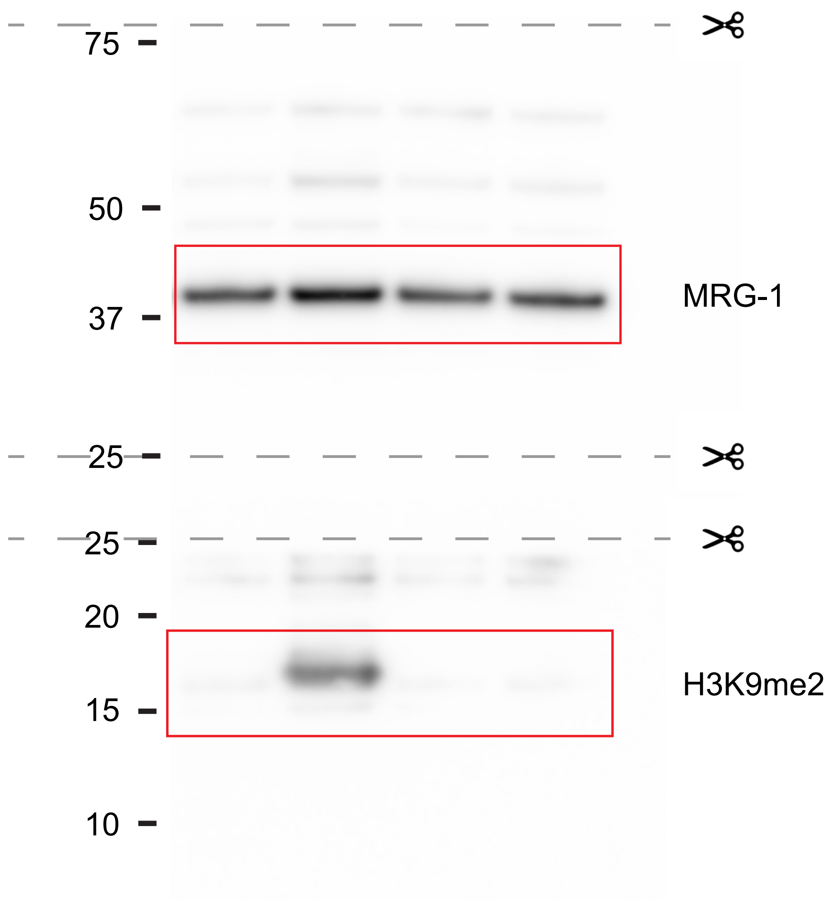

Fig. 2c

✂ = membrane cut prior to antibody incubation

Delaney et al
unprocessed blots
